# Development and validation of a radiomics-based nomogram for the prediction of postoperative malnutrition in stage IB1-IIA2 cervical carcinoma

**DOI:** 10.3389/fnut.2023.1113588

**Published:** 2023-02-03

**Authors:** Wenke Yu, Hong’en Xu, Fangjie Chen, Huafeng Shou, Ying Chen, Yongshi Jia, Hongwei Zhang, Jieni Ding, Hanchu Xiong, Yiwen Wang, Tao Song

**Affiliations:** ^1^Department of Radiology, Qingchun Hospital of Zhejiang Province, Hangzhou, China; ^2^Cancer Center, Department of Radiation Oncology, Zhejiang Provincial People’s Hospital, Affiliated People’s Hospital, Hangzhou Medical College, Hangzhou, Zhejiang, China; ^3^Department of Outpatient Nursing, Zhejiang Provincial People’s Hospital, Affiliated People’s Hospital, Hangzhou Medical College, Hangzhou, Zhejiang, China; ^4^Department of Gynecology, Zhejiang Provincial People’s Hospital, Affiliated People’s Hospital, Hangzhou Medical College, Hangzhou, Zhejiang, China; ^5^Department of Clinical Nutrition, Zhejiang Provincial People’s Hospital, Affiliated People’s Hospital, Hangzhou Medical College, Hangzhou, Zhejiang, China; ^6^Department of Clinical medical engineering, The Second Affiliated Hospital, Zhejiang University School of Medicine, Hangzhou, Zhejiang, China

**Keywords:** malnutrition, radiomics, nomogram, prediction, cervical cancer

## Abstract

**Objective:**

In individuals with stage IB1-IIA2 cervical cancer (CC) who received postoperative radiotherapy ± chemotherapy (PORT/CRT), the interaction between sarcopenia and malnutrition remains elusive, let alone employing a nomogram model based on radiomic features of psoas extracted at the level of the third lumbar vertebra (L3). This study was set to develop a radiomics-based nomogram model to predict malnutrition as per the Patient-Generated Subjective Global Assessment (PG-SGA) for individuals with CC.

**Methods:**

In total, 120 individuals with CC underwent computed tomography (CT) scans before PORT/CRT. The radiomic features of psoas at L3 were obtained from non-enhanced CT images. Identification of the optimal features and construction of the rad-score formula were conducted utilizing the least absolute shrinkage and selection operator (LASSO) logistic regression to predict malnutrition in the training dataset (radiomic model). Identification of the major clinical factors in the clinical model was performed by means of binary logistic regression analysis. The radiomics-based nomogram was further developed by integrating radiomic signatures and clinical risk factors (combined model). The receiver operating characteristic (ROC) curves and decision curves analysis (DCA) were employed for the evaluation and comparison of the three models in terms of their predictive performance.

**Results:**

Twelve radiomic features in total were chosen, and the rad-score was determined with the help of the non-zero coefficient from LASSO regression. Multivariate analysis revealed that besides rad-score, age and Eastern Cooperative Oncology Group performance status could independently predict malnutrition. As per the data of this analysis, a nomogram prediction model was constructed. The area under the ROC curves (AUC) values of the radiomic and clinical models were 0.778 and 0.847 for the training and 0.776 and 0.776 for the validation sets, respectively. An increase in the AUC was observed up to 0.972 and 0.805 in the training and validation sets, respectively, in the combined model. DCA also confirmed the clinical benefit of the combined model.

**Conclusion:**

This radiomics-based nomogram model depicted potential for use as a marker for predicting malnutrition in stage IB1-IIA2 CC patients who underwent PORT/CRT and required further investigation with a large sample size.

## Introduction

Cervical cancer (CC) remains an important health problem worldwide that is responsible for more than 600,000 new cases and 342,000 cancer-associated deaths based on the statistics of the GLOBOCAN 2020 study (1). For individuals diagnosed with International Federation of Gynecology and Obstetrics (FIGO, 2014 version) stage IB1-IIA2 CC, radical hysterectomy with lymph node dissection is the optimal therapeutic option (2). The pathological findings after surgery indicate that patients having intermediate-risk factors (such as deep stromal invasion, enlarged tumor size, or lymphatic vascular space involvement) or high-risk factors (such as positive surgical margins, lymph node metastasis, and parametrial invasion) for recurrence are recommended to receive adjuvant pelvic radiotherapy (RT) and/or platinum (cisplatin or carboplatin)-based chemoradiotherapy (CRT) to reduce the risk of tumor recurrence (3). However, around 30% of individuals with CC will still eventually develop tumor relapse, necessitating the investigation of better supportive care to improve therapeutic tolerance and reduce adverse responses in these patients (4).

Meanwhile, sarcopenia, or loss of skeletal muscle, is one of the most prevalent symptoms of malnutrition (5), and has been frequently reported as a negative factor in cancer patients at any disease stage (6, 7). In a meta-analysis, Li et al. reported that about half of the females having cancer had sarcopenia, which was significantly worse for Asian patients (8). Among the indices representing sarcopenia, the psoas parameter at the third lumbar vertebra (L3) was considered a valid indicator for identifying skeletal muscle depletion and malnutrition (9–11). In individuals with advanced lung cancer, retrospective research explored that alterations in the L3 skeletal muscle index (SMI) were consistent with the scores investigated *via* the Patient-Generated Subjective Global Assessment (PG-SGA) (12). This instrument has been considered useful for detecting malnutrition in individuals with cancer and validated on different levels (13–15). However, there are still concerns associated with patients failing to respond well to PG-SGA. In a recent surgery, Balstad and his co-authors found that an overwhelming majority of patients could complete the PG-SGA Short Form instrument properly. Though, participant- and questionnaire-linked sources of misinterpretation were still detected in some of the patients, which could lead to unfavorable results and could severely impact clinical decision-making (16, 17).

Typically, the gold standard determining skeletal muscle mass is acquired from a computed tomography (CT) scan (18) and benefits from the progress of radiomic within image processing. This study was designed to predict postoperative malnutrition assessed by means of PG-SGA with radiomic features retrieved at the psoas of L3 in individuals with FIGO stage IB1-IIA2 CC.

## Patients, materials, and methods

### Patients

Between July 2020 and June 2022, 120 patients with CC were retrospectively reviewed at the cancer center of Zhejiang Provincial People’s Hospital (ZJPPH). The eligible patients complied with the following criteria were included: (I) they underwent pelvic lymphadenectomy and radical hysterectomy and pathological diagnosis of CC; (II) they had stage IB1-IIA2 CC based on the 2014 FIGO staging system; (III) they received PORT/CRT within 1 week after admission at the ZJPPH; IV) they had Eastern Cooperative Oncology Group performance status (ECOG PS) 0–2 with no evidence of severe organ dysfunction. The exclusion criteria are mentioned below: (I) they received any anti-neoplastic treatments prior to surgery; (II) they had other malignant tumors that were contraindicated for RT; (III) poor image quality or visible artifacts around the L3 psoas. The patients’ body mass index (BMI, kg/m^2^) was adopted based on the Chinese cohort cut-off values for the identification of overweight and obesity (< 24 vs. ≥ 24) (19). The ZJPPH institutional review committee granted its approval for this study (ZJPPH No. 2022-191), and informed consent was not required.

### Treatment work-up and nutritional assessment

After surgery, patients were recommended to undergo adjuvant pelvic RT/CRT based on their pathological risk factors (3). The patients were immobilized in an immobilization device prior to PORT, and a scheduled abdomen–pelvis CT scan was routinely conducted to plan RT. The GE Discovery CT590 RT scanner was used to obtain CT scans. The main parameters are mentioned below: CT tube voltage and current were 120 kV and 250–400 mA, respectively. The thickness and spacing of the layer were both 5 mm. To eliminate any bias caused by iodinated contrast agents, non-enhanced CT images were used to derive the radiomic features.

The trained nutrition support team used the Chinese version of PG-SGA in the ward to investigate the nutritional status of the patients included in this study prior to PORT/CRT. During this research, the study subjects were classified into two groups based on previous studies (20, 21): the well-nourished group containing individuals with a PG-SGA score between 1 and 3 and the malnourished group containing individuals with a PG-SGA score ≥ 4.

### Texture feature extraction and selection

The 3D-Slicer software (v4.11, Stable Release) was used to process the non-enhanced CT images and delineate the left and right L3 psoas [volume of interest (VOI)]. This was carried out independently by one radiation oncologist (TS). Any voxel with an attenuation of < −30 or > 100 Hounsfield units was eliminated to prevent adjacent fat, bone, and surrounding organs (Supplementary Figure S1) (22).

Extraction of radiomic features was done using Pyradiomics (v3.6.2) package. In addition, 1,874 original features in total were extracted. For every VOI, 107 original, 465 Laplacian of Gaussian filter, 744 wavelet, 93 Square, 93 SquareRoot, 93 Logarithm, 93 Exponential, 93 Gradient, and 93 LocalBinaryPattern2D features were collected (Supplementary Table S1). Prior reports have mathematically defined these radiomic features (23), and these definitions can be explored at: https://pyradiomics.readthedocs.io/en/latest/features.html. After extraction, all radiomic features were subjected to further processing to conduct dimension reduction. Standardization and normalization of the features were done by means of the *Z*-score method before feature dimension reduction, thus removing unit limits from the data of every feature (24). In this study, the least absolute shrinkage and selection operator (LASSO) regression was employed to discover the most crucial features for predicting malnutrition to balance between over-fitting and under-fitting among variables (25).

The intra-observer and inter-observer agreements for feature extraction were determined with the help of the intra-class correlation coefficient (ICC) by comparing imaging data of 30 randomly selected L3 psoas from the study group (26). For the purpose of computing the intra-observer ICC, the extracted data between the two independent reader ones (TS) were compared. A second reader (HX) extraction was compared with the extraction of TS to determine the inter-observer ICC. Only the features having ICC values of ≥ 0.85 were chosen to conduct more investigations, while the rest of the segmentations were implemented by TS.

### Development of the radiomics-based nomogram

A 7:3 ratio was used to randomly classify all eligible patients into training and validation sets. Within the training set, 15% of data was applied for inter-verification. LASSO logistic regression algorithm, with penalty parameter tuning carried out by 10-fold cross-validation, was utilized to evaluate the most significant features with non-zero coefficients to predict malnutrition (PG-SGA 1–3 vs. PG-SGA ≥ 4) in the training cohort. For each patient, the radiomic feature score (rad-score) was further calculated on the basis of the LASSO binary regression model in the training cohort. The LASSO regression is formulated below:


$$\mathrm{Rad}-\mathrm{score}=\beta_{0}+\beta_{1}X_{1}+\beta_{2}X_{2}+\beta_{3}X_{3}+\ldots +\beta_{n}X_{n}$$

where *X*_1_, *X*_2_ … *X_n_* are the different radiomic features demonstrated by the LASSO, and *β*_0_ represented the intercept in the regression model. *β*_1_, *β*_2_ … *β_n_* are the regression coefficients of the corresponding features determined in the LASSO. This score was computed individually for every patient from both sets (27).

Steps for constructing the nomogram-based predictive model have been detailed in a previous study conducted by us (28). The clinical factors identified with a *p*-value of ≤ 0.05 in the univariate analysis combined with the rad-score value were incorporated into the multivariate analysis using a logistic binary regression model with a backward model selection procedure. *p* < 0.05 indicated a statistically significant level in the model. A nomogram model that incorporated the independent clinical parameters was constructed using the multivariate analysis, and the rad-score was generated for clinical reference in the training dataset.

The predictive performances of these models (radiomics, clinical, and combined models) were further evaluated both in the training and validation cohorts using the receiver operating characteristic (ROC) curves and decision curve analyses (DCAs). Calibration curves and concordance index (C-index) were utilized for the assessment of the agreement between the malnutrition probabilities predicted by the nomogram and the actual outcomes. The methods for performing univariate and multivariate binary logistic regression and comparing the area under the ROC curves (AUCs) using Delong’s test have been detailed in previous studies (29, 30).

### Statistical analysis

Python programming language (v3.7.0) was used for radiomic feature extraction and data dimension reduction. Missing data (< 5%) were processed using mean substitution (31). Further statistical analysis procedures were carried out with the help of the R software v3.6.2^1^ with the ‘*readr*, “*glmnet*, “*nomogramFormula*, “*pROC*, “*rms*, “*corrplot*’ and ‘*rmda*’ packages and the SPSS 21 (SPSS, Armonk, New York, NY, United States). Two-tailed *p* < 0.05 was considered statistically significant.

## Results

### Patients’ clinical characteristics

This study enrolled 120 individuals with FIGO stage IB1-IIA2 CC who underwent PORT/CRT. Clinical features of subjects in the training set (*n* = 84) and validation set (*n* = 36) are enlisted in Table 1. At the time of diagnosis, the median age was 54 years (interquartile range, 47–62 years), and a total of 47 (39.2%) individuals were diagnosed with malnutrition (PG-SGA score ≥ 4) with 32 (38.1%) subjects in the training and 15 (41.7%) in the validation datasets. The distribution of the baseline features across these two cohorts showed no remarkable variations.

**Table 1:** Baseline characteristics of 120 patients with FIGO stage IB1-IIA2 CC who underwent postoperative RT/CRT.

Characteristic	Frequency (*n*, %)	Training set (*n*, %)	Validation set (*n*, %)	*p* Value
*Age at diagnosis (years)*				0.098
Median (IQR)	54 (47–62)			
≤ 65	103 (85.8)	75 (89.3)	28 (77.8)	
> 65	17 (14.2)	9 (10.7)	8 (22.2)	
*ECOG PS*				0.545
0–1	91 (75.8)	65 (77.4)	26 (72.2)	
2	29 (24.2)	19 (22.6)	10 (27.8)	
*HPV infection*				0.935
Negative and unknown	46 (38.3)	32 (38.1)	14 (38.9)	
Positive	74 (61.7)	52 (61.9)	22 (61.1)	
*BMI (kg/m2)*				0.898
< 24	81 (67.5)	57 (67.9)	24 (66.7)	
≥ 24	39 (32.5)	27 (32.1)	12 (33.3)	
*PG-SGA*				0.430
1–3	77 (64.2)	52 (61.9)	25 (69.4)	
≥4	43 (35.8)	32 (38.1)	11 (30.6)	
*Histology*				
SCC	98 (81.7)	71 (84.5)	27 (75.2)	
AC and others	22 (18.3)	13 (15.5)	9 (25.0)	
*Differentiation*				0.905
Well and fairly	61 (50.8)	43 (51.2)	18 (50.0)	
Poorly and undifferentiated	59 (49.2)	41 (48.8)	18 (50.0)	
*FIGO stage*				0.300
IB	62 (51.7)	46 (54.8)	16 (44.4)	
IIA	58 (48.3)	38 (45.2)	20 (55.6)	
*Surgery approach*				0.931
Abdominal	84 (70.0)	59 (70.2)	25 (69.4)	
Laparoscopic	36 (30.0)	25 (29.8)	11 (30.6)	
*Tumor volume (mm)*				0.842
≤ 40	65 (54.2)	46 (54.8)	19 (52.8)	
> 40	55 (45.8)	38 (45.2)	17 (47.2)	
*LN metastasis*				0.412
Negative	83 (69.2)	60 (71.4)	23 (63.9)	
Positive	37 (30.8)	24 (28.6)	13 (36.1)	
*Margin*				0.164^a^
Negative	110 (91.7)	79 (94.0)	31 (96.1)	
Positive	10 (8.3)	5 (6.0)	5 (13.9)	
*Deep stromal invasion*				0.276^a^
< 1/2	9 (7.5)	8 (9.5)	1 (2.8)	
≥ 1/2	111 (92.5)	76 (90.5)	35 (97.2)	
*LVSI*				0.602
Negative	36 (30.0)	24 (28.6)	12 (33.3)	
Positive	84 (70.0)	60 (71.4)	24 (66.7)	

FIGO: International Federation of Gynecology and Obstetrics; CC: cervical cancer; CRT/RT: chemo-radiotherapy; IQR: IQR, interquartile range; ECOG PS: eastern cooperative oncology group performance status; HPV: human papillomavirus; BMI: body mass index; PG-SGA: patient-generated subjective global assessment; SCC: squamous cell carcinoma; AC: adenocarcinoma; LN: lymph node; LVSI: lymphatic vascular space involvement. ^a^Fisher’s exact test.

### Radiomic feature selection

The intra-observer ICC measured according to two extractions of reader one ranged between 0.875 and 0.932. The inter-observer agreement among two readers (TS and HX) varied from 0.837 to 0.904. Favorable intra- and inter-observer feature extraction agreements were observed in the findings.

**Figure 1:**
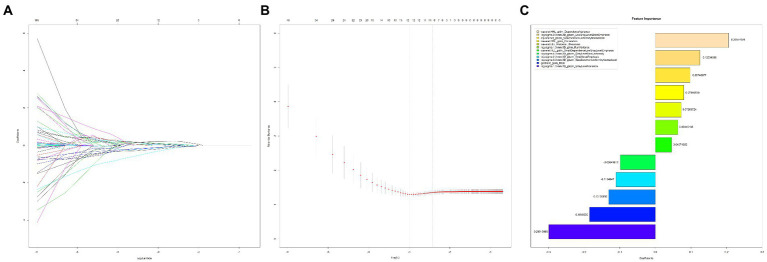
Radiomic features selection using the LASSO logistic regression model. **(A)** LASSO coefficient profiles of the 157 radiomics features. The coefficients (y-axis) were plotted against log (lambda), and the radiomics signature was constructed utilizing the selected 12 radiomic features with non-zero coefficients; **(B)**. Plotting the partial likelihood deviance against log (lambda). The upper and lower x-axis indicate the average number of predictors and the log (lambda), respectively. The y-axis denotes the partial likelihood of deviance. Utilizing the minimum criteria and one standard error of the minimum criteria, vertical lines (dotted) were created at the optimal values. The minimum criteria-based 10-fold cross-validation was utilized for the selection of the tuning parameter (λ) in the LASSO model; **(C)**. Feature importance analysis based on LASSO regression).

In the training cohort, the student’s t-test, Levene’s test, and the LASSO logistic regression analysis extracted 12 significant radiomic features with non-zero coefficients (Figures 1A,B). Further, the calculation of the rad-score was done as the sum of each feature multiplied by the non-zero coefficient from LASSO: Rad-score = −0.56187734 + 0.20554658 × wavelet.HHL_gldm_DependenceVariance +0.12534366 × log.sigma.3.0.mm.3D_glszm_LowGrayLevelZoneEmphasis + 0.09748077 × squareroot_glszm_SizeZoneNonUniformityNormalized + 0.07989539 × wavelet.HHL_glcm_Correlation + 0.07266724 × wavelet.LLL_firstorder_Skewness + 0.06345188 × log.sigma.1.0.mm.3D_glrlm_RunVariance + 0.04571352 × wavelet.HLL_gldm_SmallDependenceLowGrayLevelEmphasis + −0.09841612 × log.sigma.5.0.mm.3D_glszm_GrayLevelNonUniformity + −0.1104947 × log.sigma.2.0.mm.3D_glszm_SmallAreaEmphasis + −0.13105806 × log.sigma.3.0.mm.3D_glszm_SizeZoneNonUniformityNormalized + −0.18466055 × original_glcm_MCC + −0.29915486 × log.sigma.1.0.mm.3D_glszm_GrayLevelVariance (Figure 1C).

### Construction and performance of a rad-score-based nomogram

Univariate and multivariate binary logistic regression analyses were performed for the identification of predictive variables for malnutrition in the training set. Multivariate logistic binary regression analysis demonstrated that age [< 65 vs. ≥ 65, *p* = 0.042, odds ratio (OR) = 10.922] and ECOG PS (0–1 vs. 2, *p* = 0.008, OR = 8.672) were clinical factors significantly associated with malnutrition scored by PG-SGA (Table 2).

**Table 2:** Univariate and multivariate analyses to predict malnutrition using a binary logistic regression model.

Factor	PG-SGA							
	Univariate				Multivariate			
	*p* Value	OR	95% CI Lowers	95% CI Upper	*p* Value	OR	95% CI Lower	95% CI Upper
Age, < 65 vs. ≥ 65	0.009	17.000	2.011	143.729	0.042	10.922	1.095	108.925
ECOG PS, 0–1 vs. 2	0.001	7.311	2.300	23.244	0.008	8.672	1.765	42.608
HPV infection, No and unknown vs. Yes	0.708	0.841	0.341	2.077	-			
BMI, < 24 vs. ≥ 24	0.118	0.448	0.164	1.227	-			
Histology, SCC vs. AC and others	0.556	0.683	0.192	2.431	-			
Differentiation, well and fairly vs. poorly and undifferentiated	0.535	1.322	0.547	3.197	-			
Stage, IB vs. IIA	0.114	2.057	0.841	5.031	-			
Surgery approach, abdominal vs. laparoscopic	0.797	0.880	0.334	2.322	-			
Tumor volume, ≤ 40 vs. > 40	0.830	0.907	0.374	2.201	-			
LN metastasis, negative vs. positive	0.045	0.322	0.106	0.975	0.494	0.584	0.125	2.732
Margin, negative vs. positive	0.313	2.586	0.408	16.395	-			
Deep stromal invasion, < 1/2 vs. ≥ 1/2	0.150	0.331	0.073	1.491	-			
LVSI, negative vs. positive	0.670	0.811	0.308	2.131	-			
Radscore	< 0.001	0.112	0.040	0.317	< 0.001	0.132	0.045	0.392

OR, odds ratio; CI: confidence interval.

**Figure 2:**
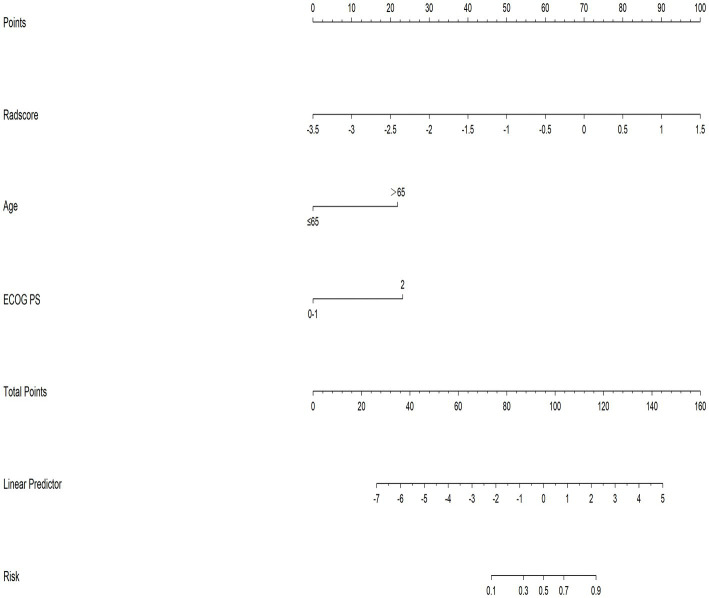
A nomogram prediction model integrated the rad-score and two clinical factors to detect malnutrition. The rad-score value is represented by the number. Every variable was located on the axis, and the corresponding point was obtained by drawing a straight line upwards to the points’ axis. The summation of all the points and identification of the location on the bottom line could be utilized to determine the estimated probability of malnutrition.

A nomogram for predicting malnutrition that integrated two clinical parameters, as demonstrated by the logistic regression and the rad-score, was further developed (Figure 2). The rad-score was regarded as the most significant prognostic parameter for malnutrition, followed by ECOG PS, and age.

The calibration plots and the C-index (0.887 for the training cohort; 0.855 for the validation cohort) revealed moderate to good agreement between the predicted and actual nutritional status between these two cohorts (Supplementary Figures S2A,B).

### Performance comparison of predictive models

**Figure 3:**
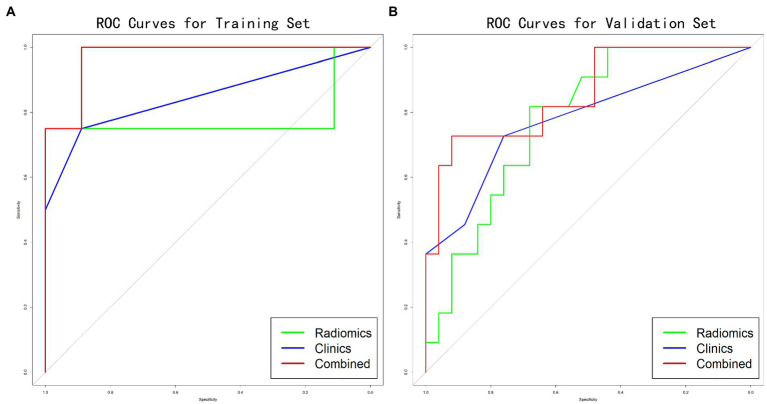
Comparing the prediction abilities of malnutrition in the training **(A)** and validation **(B)** sets. The performance of the combined prediction model (respective AUC values of  0.972 and 0.855 in the training and validation set) was greater than the radiomics or clinical model.

**Figure 4:**
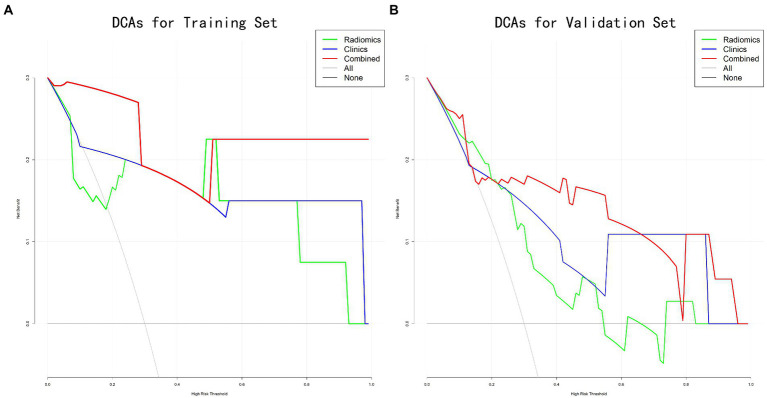
Decision curve analysis presenting the predictions of the radiomics, clinical, and combined models. **(A)** The training set; **(B)** the validation set. The threshold probability and net benefit are presented by *x*-axis and *y*-axis, respectively. The variance between the expected benefit and expected harm of the decision serves as a representation of the net benefit. The decision curve indicated that, in contrast to the other models, the combined model provided a higher net benefit.

With the training set depicting an AUC of 0.778 [95% confidence interval (CI), 0.339–1.000] and validation set depicting 0.776 AUC (95% CI, 0.623–0.930), the radiomics model revealed a moderate to good predictive efficacy. The respective AUC values of the clinical model were 0.847 (95% CI, 0.577–1.000) in the training set and 0.776 (95% CI, 0.607–0.946) in the validation set. The respective AUC values of the combined predictive model were 0.972 (95% CI, 0.895–1.000) and 0.855 (95% CI, 0.713–0.996) for the training and validation sets. Incorporating the rad-score model into the clinical model improved prediction efficacy (Figures 3A,B). The DCAs also revealed similar results indicating that the combined prediction model yielded more net benefits for predicting malnutrition than the ‘radiomics model’ and ‘clinical model’ (Figures 4A,B). However, Delong’s tests indicated no significant differences between the models.

## Discussion

This research determined the predictive capability of a radiomics-based nomogram for malnutrition in patients with FIGO stage IB1-IIA2 CC. This nomogram incorporated two readily available clinical parameters and significant radiomic features extracted from indispensable CT scans of individuals with CC who were planning to receive PORT/CRT. The results revealed that the combined prediction model incorporating significant radiomic features and clinical factors exhibited superior prediction ability compared with the other two models, indicating a considerable significance in predicting malnutrition based on medical imaging findings.

Malnutrition, as measured by PG-SGA, was reported not only to be a predictor of the high incidence of treatment-related adverse events but to have a negative impact on patient survival. Recent prospective observational research involving 391 patients with CC assessed the PG-SGA score and its link to the incidence of RT/CRT toxicity. Over half of the patients in this cohort were diagnosed with stage I-II diseases. Malnutrition was observed in 47.6% of the total population. Multivariate analysis indicated malnutrition (PG-SGA score ≥ 4) to be an independent predictor related to grade 3–4 toxicities and toxicity-associated dose modifications (32). When comparing baseline nutritional status among 207 patients with CC in Mexico, Laura et al. observed that malnutrition was an independent parameter linked to severe gastrointestinal toxicities after treatment (OR = 3.6; 95% CI, 1.46–9.2; *p* < 0.001) (33). Furthermore, a prospective study revealed that CC patients with PG-SGA score ≥ 4 had a higher mortality risk [Hazard Ratio = 3.12; 95% CI, 1.23–7.86] than patients with PG-SGA score < 4 (34).

Considering the aforementioned findings, the determination of malnutrition on the basis of objective parameters is a significant clinical challenge (15). Lee et al. conducted multiple studies in this field to demonstrate the impact of skeletal muscle loss in gynecologic patients who received RT. The skeletal muscle alterations were assessed by SMI on CT images defined at the L3 spinal level in a retrospective research involving 210 patients. The authors discovered that SMI alterations were substantially related to PG-SGA score (1–3 vs. ≥ 4) at the end of RT (*p* < 0.001). As per the multivariate analysis, PG-SGA ≥ 4 assessed at the end of RT was revealed to be an independent factor associated with increased risk of muscle loss (*p* < 0.001; OR = 72.96; 95% CI, 9.45–563.18) (35). A similar result was obtained in another observational retrospective study that enrolled 133 patients with stage IB1-IIA2 CC who received adjuvant RT/RCT. The rate of muscle loss, which was determined by SMI at the L3 vertebral level, was reported to be higher in patients with PG-SGA score ≥ 4 than in patients with PG-SGA score < 4 (71.4% vs. 2.2%, *p* < 0.001). In addition, survival analysis indicated that patients with muscle loss were significantly associated with a lower 3-year overall survival rate than patients with retained muscle (65.6 vs. 93.9%, *p* < 0.001) (36). Several other studies reported similar results regarding the association and clinical significance of skeletal muscle changes, including total adipose tissue index, SMI, skeletal muscle density, and bowel RT dose-volume (37) or distant recurrence-free survival (38) in individuals with locally advanced CC who received CRT.

Nevertheless, available literature reveals that limited studies have documented sarcopenia or malnutrition based on radiomic findings and incorporating clinical factors for nutritional prediction. A previous retrospective study from Netherlands has examined the link between radiomic features and skeletal muscle loss in 116 individuals diagnosed with stage IV non-small cell lung cancer (NSCLC). Radiomic features were also derived from CT images of the L3 vertebrae. Following feature selection, 1,298 radiomic features were extracted, and 193 of them were used to build a prediction model for muscle loss. The average AUC for radiomic features to develop the prediction model with muscle loss as the result after 100 repetitions were calculated to be only 0.49 (95% CI, 0.36–0.62). The authors concluded the inability of skeletal muscle radiomics to predict sarcopenia during chemotherapy in NSCLC (39). Kim, in contrast, expressed a different viewpoint. The radiomic features were reported to be reliable predictors of sarcopenia in patients with NSCLC by means of various machine-learning algorithms (40). Compared with the present study focused on FIGO stage IB1-IIA2 CC patients, differences in cancer types and substantial heterogeneities among patients might cause the inconsistency between the prior Dutch study (39) and the present analysis. In addition, the incorporation of significant clinical factors was carried out in an attempt to improve the prediction power, as has been done in other studies. In a retrospective study employing a radiomics-based nomogram to predict lymphovascular space invasion, 149 patients with CC undergoing surgical resection were examined, and radiomics data were collected using T2-weighted imaging. The radiomics prediction model depicted considerably better performance compared to the clinical model in both training and validation datasets. The combined nomogram prediction model incorporating radiomic features and clinical parameters yielded better performance (training cohort, AUC = 0.943; validation cohort, AUC = 0.923) than other prediction models (41). Similar studies have demonstrated that radiomics-based nomogram has robust performance in predicting lymph node metastasis (42) and survival (43) in patients with CC.

Some limitations are present in this research. First, owing to the retrospective design of the research and small sample size, prospective external validation with a large sample size is required to be conducted in the future. Second, some important parameters, such as biochemical indicators, like albumin, pre-albumin, and other clinical indicators that may potentially influence the prediction of malnutrition, were not presented. The predictive ability of the combined nomogram prediction model can be further improved. Third, there was a difference in identifying skeletal muscles at the L3 vertebral level. Some investigations have also included axial cross-sections of the skeletal muscles, which included the rectus abdominis, erector spinae, and psoas. Accuracy and repeatability in this study were guaranteed by employing the population psoas at the L3 vertebral level with an ICC of ≥ 0.85. Interestingly, Naser et al. developed a deep learning-based auto-segmentation model of cervical skeletal muscle for detecting sarcopenia in head and neck cancers (44). Several excellent nutritional risk screening instruments, except PG-SGA, were also widely employed in the clinic. These tools may provide a future direction to examine the clinical potential of a nomogram prediction model that was based on radiomics. In addition, the new Global Leadership Initiative on Malnutrition (GLIM) criteria have also revealed a promising ability to detect malnutrition and warrant further investigation (45).

## Conclusion

In summary, this study analyzed the radiomics features of psoas at the L3 level retrieved from planned CT scans of patients with FIGO stage IB1-IIA2 CC who received PORT/CRT. Furthermore, an effective and feasible nomogram prediction model on the basis of the rad-score and two clinical factors was constructed and verified for the prediction of malnutrition in patients with CC based on their PG-SGA scores. This combined nomogram prediction model presented a new strategy utilizing medical imaging data for more accurate and individualized malnutrition prediction in patients with CC, which might also be used for advanced CC and other types of malignancies in future studies.

## Data availability statement

The original contributions presented in the study are included in the article/Supplementary material, further inquiries can be directed to the corresponding author.

## Ethics statement

The studies involving human participants were reviewed and approved by the institutional review board of Zhejiang Provincial People’s Hospital (ZJPPH No. 2022-191). Written informed consent for participation was not required for this study in accordance with the national legislation and the institutional requirements.

## Author contributions

WY: conception and design, and writing original draft. HX: provision of study materials, collection data, and review of the literature. FC and HX: collection and assembly of data and writing original draft. HS and JD: collection and assembly of data and writing original draft. YC and YJ: data analysis and interpretation and writing original draft. HZ and YW: interpretation and writing original draft. TS: conception and design, provision of study materials, collection and assembly of data, drafting, final approval, and accountable for aspects. All authors contributed to the article and approved the submitted version.

## Conflict of interest

The authors declare that the research was conducted in the absence of any commercial or financial relationships that could be construed as a potential conflict of interest.

## Publisher’s note

All claims expressed in this article are solely those of the authors and do not necessarily represent those of their affiliated organizations, or those of the publisher, the editors and the reviewers. Any product that may be evaluated in this article, or claim that may be made by its manufacturer, is not guaranteed or endorsed by the publisher.

## Footnotes

^1^ https://www.r-project.org/

## Supplementary material

SUPPLEMENTARY FIGURE S1 Axial **(a)** and coronal **(b)** cross-sectional regions areas of the left and right psoas (green) on CT images at the L3 vertebral level.

SUPPLEMENTARY FIGURE S2 The calibration curves for predicting malnutrition in the **(a)** training set and **(b)** validation set, respectively. The predicted probability (from the nomogram) and the actual probability of malnutrition are represented by the x-axis and y-axis, respectively. Results were plotted via bootstrapping with 1000 resamples. Greater prediction accuracy of the model is indicated when the bias-corrected calibration curve (black line) is close to the diagonal line. The closer the line the higher the accuracy.

## References

[1] Sung H Ferlay J Siegel RL Laversanne M Soerjomataram I Jemal A. Global cancer statistics 2020: Globocan estimates of incidence and mortality worldwide for 36 cancers in 185 countries. CA Cancer J Clin. (2021) 71:209–49. doi: 10.3322/caac.21660, PMID: 33538338

[2] Cohen PA Jhingran A Oaknin A Denny L. Cervical cancer. Lancet. (2019) 393:169–82. doi: 10.1016/S0140-6736(18)32470-X 30638582

[3] Sedlis A Bundy BN Rotman MZ Lentz SS Muderspach LI Zaino RJ. A randomized trial of pelvic radiation therapy versus no further therapy in selected patients with stage Ib carcinoma of the cervix after radical hysterectomy and pelvic lymphadenectomy: a gynecologic oncology group study. Gynecol Oncol. (1999) 73:177–83. doi: 10.1006/gyno.1999.5387, PMID: 10329031

[4] Dizon DS Mackay HJ Thomas GM Werner TL Kohn EC Hess D. State of the science in cervical cancer: where we are today and where we need to go. Cancer. (2014) 120:2282–8. doi: 10.1002/cncr.28722, PMID: 24737608

[5] Thomas DR. Loss of skeletal muscle mass in aging: examining the relationship of starvation. Clin Nutr. (2007) 26:389–99. doi: 10.1016/j.clnu.2007.03.008, PMID: 17499396

[6] Chindapasirt J. Sarcopenia in cancer patients. Asian Pac J Cancer Prev. (2015) 16:8075–7. doi: 10.7314/apjcp.2015.16.18.8075 26745041

[7] Bossi P Delrio P Mascheroni A Zanetti M. The Spectrum of malnutrition/cachexia/sarcopenia in oncology according to different cancer types and settings: a narrative review. Nutrients. (2021) 13:1980. doi: 10.3390/nu13061980 34207529 PMC8226689

[8] Li YX Xia WW Liu WY. The influence process of sarcopenia on female cancer: a systematic review and meta-analysis. J Obstet Gynaecol Res. (2021) 47:4403–13. doi: 10.1111/jog.15012, PMID: 34496449

[9] Yoshikawa N Shirakawa A Yoshida K Tamauchi S Suzuki S Kikkawa F. Sarcopenia as a predictor of survival among patients with organ metastatic cervical cancer. Nutr Clin Pract. (2020) 35:1041–6. doi: 10.1002/ncp.10482, PMID: 32253779

[10] Bas O Erdemir AG Onur MR Ozer N Sener YZ Aksu S. Sarcopenia and anthracycline cardiotoxicity in patients with cancer. BMJ Support Palliat Care. (2021). doi: 10.1136/bmjspcare-2021-003197 34479960

[11] Shi B Liu S Chen J Liu J Luo Y Long L. Sarcopenia is associated with perioperative outcomes in gastric cancer patients undergoing Gastrectomy. Ann Nutr Metab. (2019) 75:213–22. doi: 10.1159/000504283, PMID: 31846973

[12] Liu X Ji W Zheng K Lu J Li L Cui J. The correlation between skeletal muscle index of the L3 vertebral body and malnutrition in patients with advanced lung cancer. BMC Cancer. (2021) 21:1148. doi: 10.1186/s12885-021-08876-4, PMID: 34702196 PMC8549206

[13] Bauer J Capra S Ferguson M. Use of the scored patient-generated subjective global assessment (Pg-Sga) as a nutrition assessment tool in patients with cancer. Eur J Clin Nutr. (2002) 56:779–85. doi: 10.1038/sj.ejcn.1601412 12122555

[14] Castillo-Martinez L Castro-Eguiluz D Copca-Mendoza ET Perez-Camargo DA Reyes-Torres CA Avila EA. Nutritional assessment tools for the identification of malnutrition and nutritional risk associated with cancer treatment. Rev Investig Clin. (2018) 70:121–5. doi: 10.24875/RIC.18002524 29943772

[15] Aprile G Basile D Giaretta R Schiavo G La Verde N Corradi E. The clinical value of nutritional care before and during active cancer treatment. Nutrients. (2021) 13:1196. doi: 10.3390/nu13041196 33916385 PMC8065908

[16] Balstad TR Bye A Jenssen CR Solheim TS Thoresen L Sand K. Patient interpretation of the patient-generated subjective global assessment (Pg-Sga) short form. Patient Prefer Adherence. (2019) 13:1391–400. doi: 10.2147/PPA.S204188, PMID: 31496666 PMC6701615

[17] Sutton RT Pincock D Baumgart DC Sadowski DC Fedorak RN Kroeker KI. An overview of clinical decision support systems: benefits, risks, and strategies for success. NPJ Digit Med. (2020) 3:17. doi: 10.1038/s41746-020-0221-y, PMID: 32047862 PMC7005290

[18] Boutin RD Yao L Canter RJ Lenchik L. Sarcopenia: current concepts and imaging implications. AJR Am J Roentgenol. (2015) 205:W255–66. doi: 10.2214/AJR.15.14635, PMID: 26102307

[19] Wang Y Mi J Shan XY Wang QJ Ge KY. Is China facing an obesity epidemic and the consequences? The trends in obesity and chronic disease in China. Int J Obes. (2007) 31:177–88. doi: 10.1038/sj.ijo.0803354, PMID: 16652128

[20] Klassen P Baracos V Gramlich L Nelson G Mazurak V Martin L. Computed-tomography body composition analysis complements pre-operative nutrition screening in colorectal cancer patients on an enhanced recovery after surgery pathway. Nutrients. (2020) 12:3745. doi: 10.3390/nu12123745 33291416 PMC7762071

[21] Ho CY Ibrahim Z Abu Zaid Z Mat Daud Z Md Yusop NB. Clinical malnutrition predictive model among gynecologic cancer patients prior to elective operation: a cross-sectional study. Clin Nutr. (2021) 40:4373–9. doi: 10.1016/j.clnu.2021.01.008, PMID: 33485706

[22] Mitsiopoulos N Baumgartner RN Heymsfield SB Lyons W Gallagher D Ross R. Cadaver validation of skeletal muscle measurement by magnetic resonance imaging and computerized tomography. J Appl Physiol. (1985) 1998:115–22. doi: 10.1152/jappl.1998.85.1.115 9655763

[23] van Griethuysen JJM Fedorov A Parmar C Hosny A Aucoin N Narayan V. Computational Radiomics system to decode the radiographic phenotype. Cancer Res. (2017) 77:e104–7. doi: 10.1158/0008-5472.CAN-17-0339, PMID: 29092951 PMC5672828

[24] Cheadle C Vawter MP Freed WJ Becker KG. Analysis of microarray data using Z score transformation. J Mol Diagn. (2003) 5:73–81. doi: 10.1016/S1525-1578(10)60455-2, PMID: 12707371 PMC1907322

[25] Li Z Sillanpaa MJ. Overview of Lasso-related penalized regression methods for quantitative trait mapping and genomic selection. Theor Appl Genet. (2012) 125:419–35. doi: 10.1007/s00122-012-1892-9, PMID: 22622521

[26] Nakagawa S Johnson PCD Schielzeth H. The coefficient of determination R(2) and intra-class correlation coefficient from generalized linear mixed-effects models revisited and expanded. J R Soc Interface. (2017) 14:20170213. doi: 10.1098/rsif.2017.0213 28904005 PMC5636267

[27] Luo HS Chen YY Huang WZ Wu SX Huang SF Xu HY. Development and validation of a Radiomics-based model to predict local progression-free survival after chemo-radiotherapy in patients with esophageal squamous cell cancer. Radiat Oncol. (2021) 16:201. doi: 10.1186/s13014-021-01925-z, PMID: 34641928 PMC8513312

[28] Yu W Huang L Zhong Z Song T Xu H Jia Y. A Nomogram-based risk classification system predicting the overall survival of patients with newly diagnosed stage Ivb cervix uteri carcinoma. Front Med (Lausanne). (2021) 8:693567. doi: 10.3389/fmed.2021.693567, PMID: 34336897 PMC8319470

[29] Song T Chen L Zhang H Lu Y Yu K Zhan W. Multimodal treatment based on thyroidectomy improves survival in patients with metastatic anaplastic thyroid carcinoma: a seer analysis from 1998 to 2015. Gland Surg. (2020) 9:1205–13. doi: 10.21037/gs-20-503 33224795 PMC7667089

[30] Song T Xu H Shi L Yan S. Prognostic analysis and comparison of the 2014 and 2018 International Federation of Gynecology and Obstetrics Staging System on overall survival in patients with stage Iib-Iva cervix carcinoma. Int J Women's Health. (2022) 14:333–44. doi: 10.2147/IJWH.S348074, PMID: 35283649 PMC8909488

[31] Ali AM Dawson SJ Blows FM Provenzano E Ellis IO Baglietto L. Comparison of methods for handling missing data on immunohistochemical markers in survival analysis of breast cancer. Br J Cancer. (2011) 104:693–9. doi: 10.1038/sj.bjc.6606078, PMID: 21266980 PMC3049587

[32] Mota AP Aredes MA De Oliveira MJ Chaves GV. Nutritional status assessed by patient-generated subjective global assessment is associated with toxicity to chemoradiotherapy in women with cervical cancer: a prospective study. Eur J Clin Nutr. (2022) 76:1740–7. doi: 10.1038/s41430-022-01180-9 35854132

[33] Laura FC Lucely CP Tatiana GC Roberto JL Dulce GI Arturo PS. Handgrip strength, Overhydration and nutritional status as a predictors of gastrointestinal toxicity in cervical cancer patients. A prospective study. Nutr Cancer. (2022) 74:2444–50. doi: 10.1080/01635581.2021.2012209, PMID: 35023398

[34] Argefa TG Roets L. Malnutrition and the survival of cervical cancer patients: a prospective cohort study using the Pg-Sga tool. Nutr Cancer. (2022) 74:605–12. doi: 10.1080/01635581.2021.1910320, PMID: 33899611

[35] Lee J Chen TC Jan YT Li CJ Chen YJ Wu MH. Association of patient-reported outcomes and nutrition with body composition in women with gynecologic cancer undergoing post-operative pelvic radiotherapy: An observational study. Nutrients. (2021) 13:2629. doi: 10.3390/nu13082629 34444789 PMC8399258

[36] Lee J Lin JB Chen TC Jan YT Sun FJ Chen YJ. Progressive skeletal muscle loss after surgery and adjuvant radiotherapy impact survival outcomes in patients with early stage cervical cancer. Front Nutr. (2021) 8:773506. doi: 10.3389/fnut.2021.773506, PMID: 35127782 PMC8810512

[37] Lee J Lin JB Wu MH Chang CL Jan YT Sun FJ. Association of Bowel Radiation Dose-Volume with skeletal muscle loss during pelvic intensity-modulated radiotherapy in cervical cancer. Support Care Cancer. (2021) 29:5497–505. Epub 2021/03/14. doi: 10.1007/s00520-021-06131-x, PMID: 33712910

[38] Lee J Lin JB Wu MH Chang CL Jan YT Chen YJ. Muscle loss after Chemoradiotherapy as a biomarker of distant failures in locally advanced cervical cancer. Cancers (Basel). (2020) 12:595. doi: 10.3390/cancers12030595 32150938 PMC7139727

[39] de Jong EEC Sanders KJC Deist TM van Elmpt W Jochems A van Timmeren JE. Can Radiomics help to predict skeletal muscle response to chemotherapy in stage iv non-small cell lung cancer? Eur J Cancer. (2019) 120:107–13. doi: 10.1016/j.ejca.2019.07.023, PMID: 31514107

[40] Kim YJ. Machine learning models for sarcopenia identification based on Radiomic features of muscles in computed tomography. Int J Environ Res Public Health. (2021) 18:8710. doi: 10.3390/ijerph18168710 34444459 PMC8394435

[41] Du W Wang Y Li D Xia X Tan Q Xiong X. Preoperative prediction of lymphovascular space invasion in cervical cancer with radiomics-based nomogram. Front Oncol. (2021) 11:637794. doi: 10.3389/fonc.2021.637794, PMID: 34322375 PMC8311659

[42] Hou L Zhou W Ren J Du X Xin L Zhao X. Radiomics analysis of multiparametric Mri for the preoperative prediction of lymph node metastasis in cervical cancer. Front Oncol. (2020) 10:1393. doi: 10.3389/fonc.2020.01393, PMID: 32974143 PMC7468409

[43] Zhang X Zhao J Zhang Q Wang S Zhang J An J. MRI-based radiomics value for predicting the survival of patients with locally advanced cervical squamous cell cancer treated with concurrent chemoradiotherapy. Cancer Imaging. (2022) 22:35. doi: 10.1186/s40644-022-00474-2, PMID: 35842679 PMC9287951

[44] Naser MA Wahid KA Grossberg AJ Olson B Jain R El-Habashy D. Deep learning auto-segmentation of cervical skeletal muscle for sarcopenia analysis in patients with head and neck cancer. Front Oncol. (2022) 12:930432. doi: 10.3389/fonc.2022.930432, PMID: 35965493 PMC9366009

[45] De Groot LM Lee G Ackerie A van der Meij BS. Malnutrition screening and assessment in the cancer care ambulatory setting: mortality predictability and validity of the patient-generated subjective global assessment short form (Pg-Sga Sf) and the Glim criteria. Nutrients. (2020) 12:2287. doi: 10.3390/nu12082287 32751724 PMC7468976

