# Supplementary figures and images for: Development and validation of a radiomics-based nomogram for the prediction of postoperative malnutrition in stage IB1-IIA2 cervical carcinoma
Source: Front Nutr. 2023 Feb 3;10:1113588. doi: 10.3389/fnut.2023.1113588 (PMC9936189; doi:10.3389/fnut.2023.1113588)

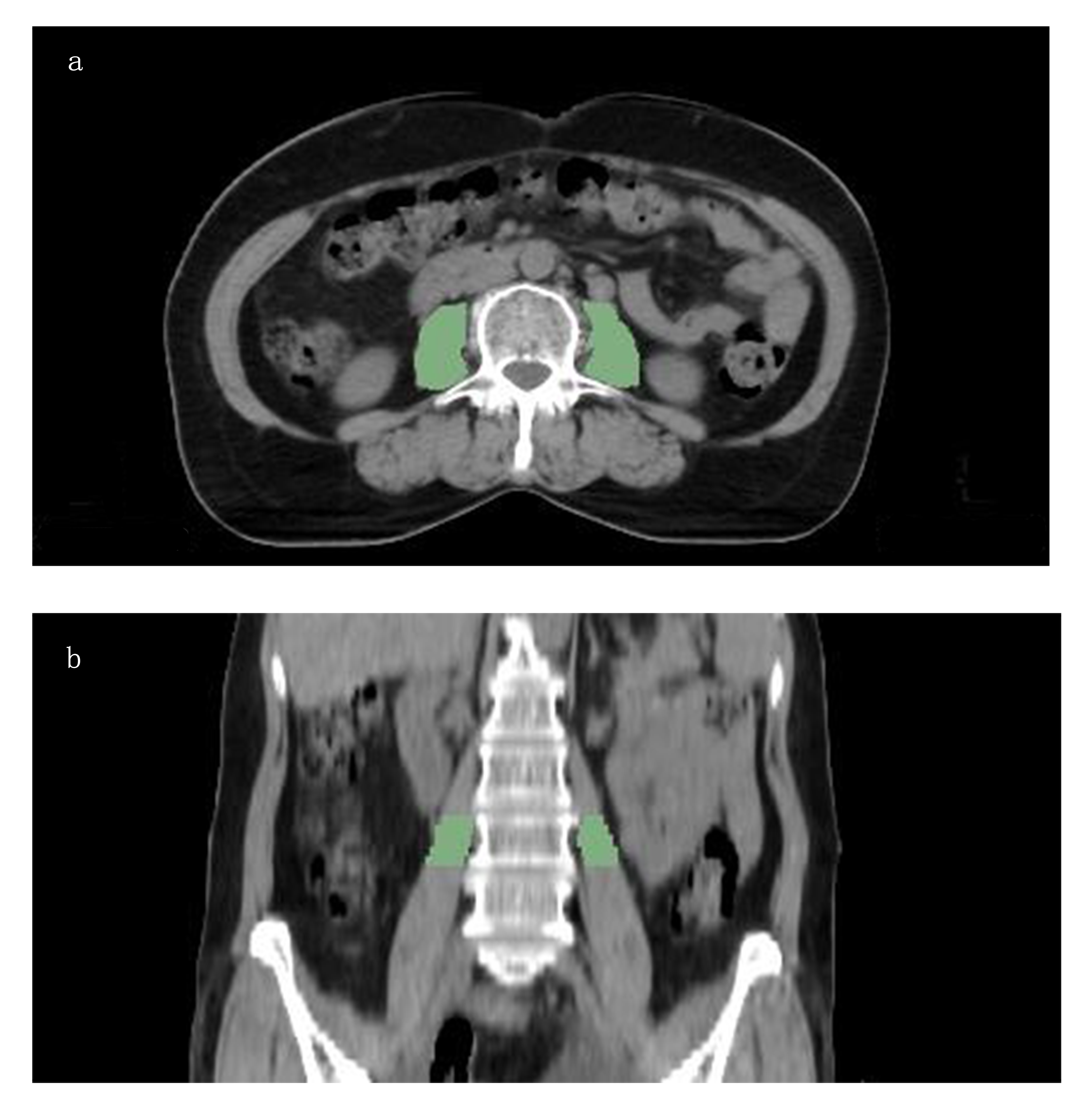

Supplement: SUPPLEMENTARY FIGURE S1 — Axial (a) and coronal (b) cross-sectional regions areas of the left and right psoas (green) on CT images at the L3 vertebral level. [file Image_1.TIF]

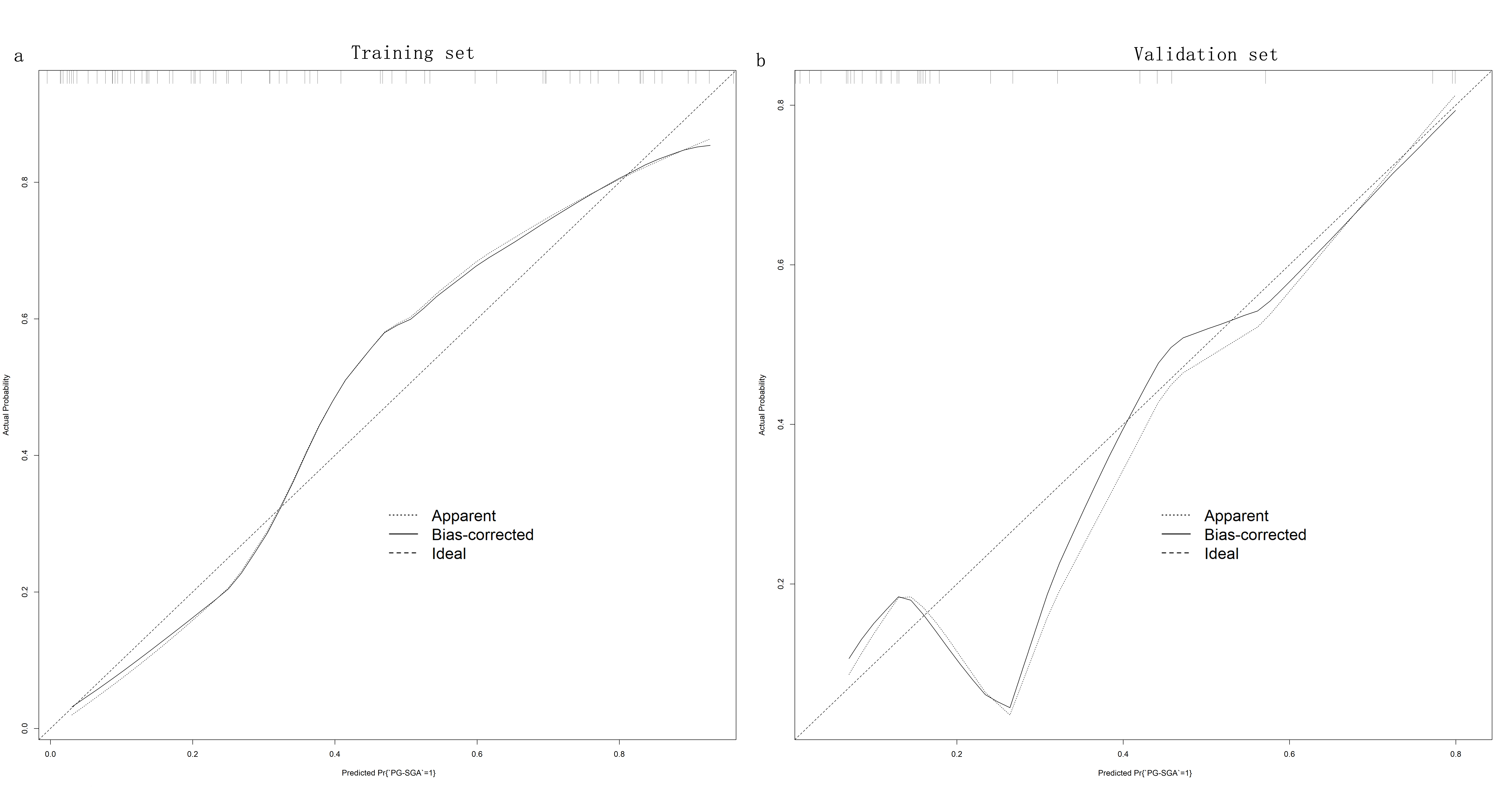

Supplement: SUPPLEMENTARY FIGURE S2 — The calibration curves for predicting malnutrition in the (a) training set and (b) validation set, respectively. The predicted probability (from the nomogram) and the actual probability of malnutrition are represented by the x-axis and y-axis, respectively. Results were plotted via bootstrapping with 1000 resamples. Greater prediction accuracy of the model is indicated when the bias-corrected calibration curve (black line) is close to the diagonal line. The closer the line the higher the accuracy. [file Image_2.TIF]
