# Supplementary material for: Development and validation of a radiomics-based nomogram for the prediction of postoperative malnutrition in stage IB1-IIA2 cervical carcinoma
Source: Front Nutr. 2023 Feb 3;10:1113588. doi: 10.3389/fnut.2023.1113588 (PMC9936189; doi:10.3389/fnut.2023.1113588)
Supplement: Supplementary file 3 [file Table_1.DOCX]

Table S1. The list of radiomic features extracted from the VOIs.

|  | Image type | Feature | Feature name |
| --- | --- | --- | --- |
| F1 | Original | Shape | Elongation |
| F2 |  | Shape | Flatness |
| F3 |  | Shape | LeastAxisLength |
| F4 |  | Shape | MajorAxisLength |
| F5 |  | Shape | Maximum2DDiameterColumn |
| F6 |  | Shape | Maximum2DDiameterRow |
| F7 |  | Shape | Maximum2DDiameterSlice |
| F8 |  | Shape | Maximum3DDiameter |
| F9 |  | Shape | MeshVolume |
| F10 |  | Shape | MinorAxisLength |
| F11 |  | Shape | Sphericity |
| F12 |  | Shape | SurfaceArea |
| F13 |  | Shape | SurfaceVolumeRatio |
| F14 |  | Shape | VoxelVolume |
| F15 |  | Firstorder | 10Percentile |
| F16 |  | Firstorder | 90Percentile |
| F17 |  | Firstorder | Energy |
| F18 |  | Firstorder | Entropy |
| F19 |  | Firstorder | InterquartileRange |
| F20 |  | Firstorder | Kurtosis |
| F21 |  | Firstorder | Maximum |
| F22 |  | Firstorder | MeanAbsoluteDeviation |
| F23 |  | Firstorder | Mean |
| F24 |  | Firstorder | Median |
| F25 |  | Firstorder | Minimum |
| F26 |  | Firstorder | Range |
| F27 |  | Firstorder | RobustMeanAbsoluteDeviation |
| F28 |  | Firstorder | RootMeanSquared |
| F29 |  | Firstorder | Skewness |
| F30 |  | Firstorder | TotalEnergy |
| F31 |  | Firstorder | Uniformity |
| F32 |  | Firstorder | Variance |
| F33 |  | GLCM | Autocorrelation |
| F34 |  | GLCM | ClusterProminence |
| F35 |  | GLCM | ClusterShade |
| F36 |  | GLCM | ClusterTendency |
| F37 |  | GLCM | Contrast |
| F38 |  | GLCM | Correlation |
| F39 |  | GLCM | DifferenceAverage |
| F40 |  | GLCM | DifferenceEntropy |
| F41 |  | GLCM | DifferenceVariance |
| F42 |  | GLCM | Id |
| F43 |  | GLCM | Idm |
| F44 |  | GLCM | Idmn |
| F45 |  | GLCM | Idn |
| F46 |  | GLCM | Imc1 |
| F47 |  | GLCM | Imc2 |
| F48 |  | GLCM | InverseVariance |
| F49 |  | GLCM | JointAverage |
| F50 |  | GLCM | JointEnergy |
| F51 |  | GLCM | JointEntropy |
| F52 |  | GLCM | MCC |
| F53 |  | GLCM | MaximumProbability |
| F54 |  | GLCM | SumAverage |
| F55 |  | GLCM | SumEntropy |
| F56 |  | GLCM | SumSquares |
| F57 |  | GLRLM | GrayLevelNonUniformity |
| F58 |  | GLRLM | GrayLevelNonUniformityNormalized |
| F59 |  | GLRLM | GrayLevelVariance |
| F60 |  | GLRLM | HighGrayLevelRunEmphasis |
| F61 |  | GLRLM | LongRunEmphasis |
| F62 |  | GLRLM | LongRunHighGrayLevelEmphasis |
| F63 |  | GLRLM | LongRunLowGrayLevelEmphasis |
| F64 |  | GLRLM | LowGrayLevelRunEmphasis |
| F65 |  | GLRLM | RunEntropy |
| F66 |  | GLRLM | RunLengthNonUniformity |
| F67 |  | GLRLM | RunLengthNonUniformityNormalized |
| F68 |  | GLRLM | RunPercentage |
| F69 |  | GLRLM | RunVariance |
| F70 |  | GLRLM | ShortRunEmphasis |
| F71 |  | GLRLM | ShortRunHighGrayLevelEmphasis |
| F72 |  | GLRLM | ShortRunLowGrayLevelEmphasis |
| F73 |  | GLSZM | GrayLevelNonUniformity |
| F74 |  | GLSZM | GrayLevelNonUniformityNormalized |
| F75 |  | GLSZM | GrayLevelVariance |
| F76 |  | GLSZM | HighGrayLevelZoneEmphasis |
| F77 |  | GLSZM | LargeAreaEmphasis |
| F78 |  | GLSZM | LargeAreaHighGrayLevelEmphasis |
| F79 |  | GLSZM | LargeAreaLowGrayLevelEmphasis |
| F80 |  | GLSZM | LowGrayLevelZoneEmphasis |
| F81 |  | GLSZM | SizeZoneNonUniformity |
| F82 |  | GLSZM | SizeZoneNonUniformityNormalized |
| F83 |  | GLSZM | SmallAreaEmphasis |
| F84 |  | GLSZM | SmallAreaHighGrayLevelEmphasis |
| F85 |  | GLSZM | SmallAreaLowGrayLevelEmphasis |
| F86 |  | GLSZM | ZoneEntropy |
| F87 |  | GLSZM | ZonePercentage |
| F88 |  | GLSZM | ZoneVariance |
| F89 |  | GLDM | DependenceEntropy |
| F90 |  | GLDM | DependenceNonUniformity |
| F91 |  | GLDM | DependenceNonUniformityNormalized |
| F92 |  | GLDM | DependenceVariance |
| F93 |  | GLDM | GrayLevelNonUniformity |
| F94 |  | GLDM | GrayLevelVariance |
| F95 |  | GLDM | HighGrayLevelEmphasis |
| F96 |  | GLDM | LargeDependenceEmphasis |
| F97 |  | GLDM | LargeDependenceHighGrayLevelEmphasis |
| F98 |  | GLDM | LargeDependenceLowGrayLevelEmphasis |
| F99 |  | GLDM | LowGrayLevelEmphasis |
| F100 |  | GLDM | SmallDependenceEmphasis |
| F101 |  | GLDM | SmallDependenceHighGrayLevelEmphasis |
| F102 |  | GLDM | SmallDependenceLowGrayLevelEmphasis |
| F103 |  | NGTDM | Busyness |
| F104 |  | NGTDM | Coarseness |
| F105 |  | NGTDM | Complexity |
| F106 |  | NGTDM | Contrast |
| F107 |  | NGTDM | Strength |
| F108 | LoG (sigma.1.0.mm.3D) | Firstorder | 10Percentile |
| F109 |  | Firstorder | 90Percentile |
| F110 |  | Firstorder | Energy |
| F111 |  | Firstorder | Entropy |
| F112 |  | Firstorder | InterquartileRange |
| F113 |  | Firstorder | Kurtosis |
| F114 |  | Firstorder | Maximum |
| F115 |  | Firstorder | MeanAbsoluteDeviation |
| F116 |  | Firstorder | Mean |
| F117 |  | Firstorder | Median |
| F118 |  | Firstorder | Minimum |
| F119 |  | Firstorder | Range |
| F120 |  | Firstorder | RobustMeanAbsoluteDeviation |
| F121 |  | Firstorder | RootMeanSquared |
| F122 |  | Firstorder | Skewness |
| F123 |  | Firstorder | TotalEnergy |
| F124 |  | Firstorder | Uniformity |
| F125 |  | Firstorder | Variance |
| F126 |  | GLCM | Autocorrelation |
| F127 |  | GLCM | ClusterProminence |
| F128 |  | GLCM | ClusterShade |
| F129 |  | GLCM | ClusterTendency |
| F130 |  | GLCM | Contrast |
| F131 |  | GLCM | Correlation |
| F132 |  | GLCM | DifferenceAverage |
| F133 |  | GLCM | DifferenceEntropy |
| F134 |  | GLCM | DifferenceVariance |
| F135 |  | GLCM | Id |
| F136 |  | GLCM | Idm |
| F137 |  | GLCM | Idmn |
| F138 |  | GLCM | Idn |
| F139 |  | GLCM | Imc1 |
| F140 |  | GLCM | Imc2 |
| F141 |  | GLCM | InverseVariance |
| F142 |  | GLCM | JointAverage |
| F143 |  | GLCM | JointEnergy |
| F144 |  | GLCM | JointEntropy |
| F145 |  | GLCM | MCC |
| F146 |  | GLCM | MaximumProbability |
| F147 |  | GLCM | SumAverage |
| F148 |  | GLCM | SumEntropy |
| F149 |  | GLCM | SumSquares |
| F150 |  | GLRLM | GrayLevelNonUniformity |
| F151 |  | GLRLM | GrayLevelNonUniformityNormalized |
| F152 |  | GLRLM | GrayLevelVariance |
| F153 |  | GLRLM | HighGrayLevelRunEmphasis |
| F154 |  | GLRLM | LongRunEmphasis |
| F155 |  | GLRLM | LongRunHighGrayLevelEmphasis |
| F156 |  | GLRLM | LongRunLowGrayLevelEmphasis |
| F157 |  | GLRLM | LowGrayLevelRunEmphasis |
| F158 |  | GLRLM | RunEntropy |
| F159 |  | GLRLM | RunLengthNonUniformity |
| F160 |  | GLRLM | RunLengthNonUniformityNormalized |
| F161 |  | GLRLM | RunPercentage |
| F162 |  | GLRLM | RunVariance |
| F163 |  | GLRLM | ShortRunEmphasis |
| F164 |  | GLRLM | ShortRunHighGrayLevelEmphasis |
| F165 |  | GLRLM | ShortRunLowGrayLevelEmphasis |
| F166 |  | GLSZM | GrayLevelNonUniformity |
| F167 |  | GLSZM | GrayLevelNonUniformityNormalized |
| F168 |  | GLSZM | GrayLevelVariance |
| F169 |  | GLSZM | HighGrayLevelZoneEmphasis |
| F170 |  | GLSZM | LargeAreaEmphasis |
| F171 |  | GLSZM | LargeAreaHighGrayLevelEmphasis |
| F172 |  | GLSZM | LargeAreaLowGrayLevelEmphasis |
| F173 |  | GLSZM | LowGrayLevelZoneEmphasis |
| F174 |  | GLSZM | SizeZoneNonUniformity |
| F175 |  | GLSZM | SizeZoneNonUniformityNormalized |
| F176 |  | GLSZM | SmallAreaEmphasis |
| F177 |  | GLSZM | SmallAreaHighGrayLevelEmphasis |
| F178 |  | GLSZM | SmallAreaLowGrayLevelEmphasis |
| F179 |  | GLSZM | ZoneEntropy |
| F180 |  | GLSZM | ZonePercentage |
| F181 |  | GLSZM | ZoneVariance |
| F182 |  | GLDM | DependenceEntropy |
| F183 |  | GLDM | DependenceNonUniformity |
| F184 |  | GLDM | DependenceNonUniformityNormalized |
| F185 |  | GLDM | DependenceVariance |
| F186 |  | GLDM | GrayLevelNonUniformity |
| F187 |  | GLDM | GrayLevelVariance |
| F188 |  | GLDM | HighGrayLevelEmphasis |
| F189 |  | GLDM | LargeDependenceEmphasis |
| F190 |  | GLDM | LargeDependenceHighGrayLevelEmphasis |
| F191 |  | GLDM | LargeDependenceLowGrayLevelEmphasis |
| F192 |  | GLDM | LowGrayLevelEmphasis |
| F193 |  | GLDM | SmallDependenceEmphasis |
| F194 |  | GLDM | SmallDependenceHighGrayLevelEmphasis |
| F195 |  | GLDM | SmallDependenceLowGrayLevelEmphasis |
| F196 |  | NGTDM | Busyness |
| F197 |  | NGTDM | Coarseness |
| F198 |  | NGTDM | Complexity |
| F199 |  | NGTDM | Contrast |
| F200 |  | NGTDM | Strength |
| F201 | LoG (sigma.2.0.mm.3D) | Firstorder | 10Percentile |
| F202 |  | Firstorder | 90Percentile |
| F203 |  | Firstorder | Energy |
| F204 |  | Firstorder | Entropy |
| F205 |  | Firstorder | InterquartileRange |
| F206 |  | Firstorder | Kurtosis |
| F207 |  | Firstorder | Maximum |
| F208 |  | Firstorder | MeanAbsoluteDeviation |
| F209 |  | Firstorder | Mean |
| F210 |  | Firstorder | Median |
| F211 |  | Firstorder | Minimum |
| F212 |  | Firstorder | Range |
| F213 |  | Firstorder | RobustMeanAbsoluteDeviation |
| F214 |  | Firstorder | RootMeanSquared |
| F215 |  | Firstorder | Skewness |
| F216 |  | Firstorder | TotalEnergy |
| F217 |  | Firstorder | Uniformity |
| F218 |  | Firstorder | Variance |
| F219 |  | GLCM | Autocorrelation |
| F220 |  | GLCM | ClusterProminence |
| F221 |  | GLCM | ClusterShade |
| F222 |  | GLCM | ClusterTendency |
| F223 |  | GLCM | Contrast |
| F224 |  | GLCM | Correlation |
| F225 |  | GLCM | DifferenceAverage |
| F226 |  | GLCM | DifferenceEntropy |
| F227 |  | GLCM | DifferenceVariance |
| F228 |  | GLCM | Id |
| F229 |  | GLCM | Idm |
| F230 |  | GLCM | Idmn |
| F231 |  | GLCM | Idn |
| F232 |  | GLCM | Imc1 |
| F233 |  | GLCM | Imc2 |
| F234 |  | GLCM | InverseVariance |
| F235 |  | GLCM | JointAverage |
| F236 |  | GLCM | JointEnergy |
| F237 |  | GLCM | JointEntropy |
| F238 |  | GLCM | MCC |
| F239 |  | GLCM | MaximumProbability |
| F240 |  | GLCM | SumAverage |
| F241 |  | GLCM | SumEntropy |
| F242 |  | GLCM | SumSquares |
| F243 |  | GLRLM | GrayLevelNonUniformity |
| F244 |  | GLRLM | GrayLevelNonUniformityNormalized |
| F245 |  | GLRLM | GrayLevelVariance |
| F246 |  | GLRLM | HighGrayLevelRunEmphasis |
| F247 |  | GLRLM | LongRunEmphasis |
| F248 |  | GLRLM | LongRunHighGrayLevelEmphasis |
| F249 |  | GLRLM | LongRunLowGrayLevelEmphasis |
| F250 |  | GLRLM | LowGrayLevelRunEmphasis |
| F251 |  | GLRLM | RunEntropy |
| F252 |  | GLRLM | RunLengthNonUniformity |
| F253 |  | GLRLM | RunLengthNonUniformityNormalized |
| F254 |  | GLRLM | RunPercentage |
| F255 |  | GLRLM | RunVariance |
| F256 |  | GLRLM | ShortRunEmphasis |
| F257 |  | GLRLM | ShortRunHighGrayLevelEmphasis |
| F258 |  | GLRLM | ShortRunLowGrayLevelEmphasis |
| F259 |  | GLSZM | GrayLevelNonUniformity |
| F260 |  | GLSZM | GrayLevelNonUniformityNormalized |
| F261 |  | GLSZM | GrayLevelVariance |
| F262 |  | GLSZM | HighGrayLevelZoneEmphasis |
| F263 |  | GLSZM | LargeAreaEmphasis |
| F264 |  | GLSZM | LargeAreaHighGrayLevelEmphasis |
| F265 |  | GLSZM | LargeAreaLowGrayLevelEmphasis |
| F266 |  | GLSZM | LowGrayLevelZoneEmphasis |
| F267 |  | GLSZM | SizeZoneNonUniformity |
| F268 |  | GLSZM | SizeZoneNonUniformityNormalized |
| F269 |  | GLSZM | SmallAreaEmphasis |
| F270 |  | GLSZM | SmallAreaHighGrayLevelEmphasis |
| F271 |  | GLSZM | SmallAreaLowGrayLevelEmphasis |
| F272 |  | GLSZM | ZoneEntropy |
| F273 |  | GLSZM | ZonePercentage |
| F274 |  | GLSZM | ZoneVariance |
| F275 |  | GLDM | DependenceEntropy |
| F276 |  | GLDM | DependenceNonUniformity |
| F277 |  | GLDM | DependenceNonUniformityNormalized |
| F278 |  | GLDM | DependenceVariance |
| F279 |  | GLDM | GrayLevelNonUniformity |
| F280 |  | GLDM | GrayLevelVariance |
| F281 |  | GLDM | HighGrayLevelEmphasis |
| F282 |  | GLDM | LargeDependenceEmphasis |
| F283 |  | GLDM | LargeDependenceHighGrayLevelEmphasis |
| F284 |  | GLDM | LargeDependenceLowGrayLevelEmphasis |
| F285 |  | GLDM | LowGrayLevelEmphasis |
| F286 |  | GLDM | SmallDependenceEmphasis |
| F287 |  | GLDM | SmallDependenceHighGrayLevelEmphasis |
| F288 |  | GLDM | SmallDependenceLowGrayLevelEmphasis |
| F289 |  | NGTDM | Busyness |
| F290 |  | NGTDM | Coarseness |
| F291 |  | NGTDM | Complexity |
| F292 |  | NGTDM | Contrast |
| F293 |  | NGTDM | Strength |
| F294 | LoG (sigma.3.0.mm.3D) | Firstorder | 10Percentile |
| F295 |  | Firstorder | 90Percentile |
| F296 |  | Firstorder | Energy |
| F297 |  | Firstorder | Entropy |
| F298 |  | Firstorder | InterquartileRange |
| F299 |  | Firstorder | Kurtosis |
| F300 |  | Firstorder | Maximum |
| F301 |  | Firstorder | MeanAbsoluteDeviation |
| F302 |  | Firstorder | Mean |
| F303 |  | Firstorder | Median |
| F304 |  | Firstorder | Minimum |
| F305 |  | Firstorder | Range |
| F306 |  | Firstorder | RobustMeanAbsoluteDeviation |
| F307 |  | Firstorder | RootMeanSquared |
| F308 |  | Firstorder | Skewness |
| F309 |  | Firstorder | TotalEnergy |
| F310 |  | Firstorder | Uniformity |
| F311 |  | Firstorder | Variance |
| F312 |  | GLCM | Autocorrelation |
| F313 |  | GLCM | ClusterProminence |
| F314 |  | GLCM | ClusterShade |
| F315 |  | GLCM | ClusterTendency |
| F316 |  | GLCM | Contrast |
| F317 |  | GLCM | Correlation |
| F318 |  | GLCM | DifferenceAverage |
| F319 |  | GLCM | DifferenceEntropy |
| F320 |  | GLCM | DifferenceVariance |
| F321 |  | GLCM | Id |
| F322 |  | GLCM | Idm |
| F323 |  | GLCM | Idmn |
| F324 |  | GLCM | Idn |
| F325 |  | GLCM | Imc1 |
| F326 |  | GLCM | Imc2 |
| F327 |  | GLCM | InverseVariance |
| F328 |  | GLCM | JointAverage |
| F329 |  | GLCM | JointEnergy |
| F330 |  | GLCM | JointEntropy |
| F331 |  | GLCM | MCC |
| F332 |  | GLCM | MaximumProbability |
| F333 |  | GLCM | SumAverage |
| F334 |  | GLCM | SumEntropy |
| F335 |  | GLCM | SumSquares |
| F336 |  | GLRLM | GrayLevelNonUniformity |
| F337 |  | GLRLM | GrayLevelNonUniformityNormalized |
| F338 |  | GLRLM | GrayLevelVariance |
| F339 |  | GLRLM | HighGrayLevelRunEmphasis |
| F340 |  | GLRLM | LongRunEmphasis |
| F341 |  | GLRLM | LongRunHighGrayLevelEmphasis |
| F342 |  | GLRLM | LongRunLowGrayLevelEmphasis |
| F343 |  | GLRLM | LowGrayLevelRunEmphasis |
| F344 |  | GLRLM | RunEntropy |
| F345 |  | GLRLM | RunLengthNonUniformity |
| F346 |  | GLRLM | RunLengthNonUniformityNormalized |
| F347 |  | GLRLM | RunPercentage |
| F348 |  | GLRLM | RunVariance |
| F349 |  | GLRLM | ShortRunEmphasis |
| F350 |  | GLRLM | ShortRunHighGrayLevelEmphasis |
| F351 |  | GLRLM | ShortRunLowGrayLevelEmphasis |
| F352 |  | GLSZM | GrayLevelNonUniformity |
| F353 |  | GLSZM | GrayLevelNonUniformityNormalized |
| F354 |  | GLSZM | GrayLevelVariance |
| F355 |  | GLSZM | HighGrayLevelZoneEmphasis |
| F356 |  | GLSZM | LargeAreaEmphasis |
| F357 |  | GLSZM | LargeAreaHighGrayLevelEmphasis |
| F358 |  | GLSZM | LargeAreaLowGrayLevelEmphasis |
| F359 |  | GLSZM | LowGrayLevelZoneEmphasis |
| F360 |  | GLSZM | SizeZoneNonUniformity |
| F361 |  | GLSZM | SizeZoneNonUniformityNormalized |
| F362 |  | GLSZM | SmallAreaEmphasis |
| F363 |  | GLSZM | SmallAreaHighGrayLevelEmphasis |
| F364 |  | GLSZM | SmallAreaLowGrayLevelEmphasis |
| F365 |  | GLSZM | ZoneEntropy |
| F366 |  | GLSZM | ZonePercentage |
| F367 |  | GLSZM | ZoneVariance |
| F368 |  | GLDM | DependenceEntropy |
| F369 |  | GLDM | DependenceNonUniformity |
| F370 |  | GLDM | DependenceNonUniformityNormalized |
| F371 |  | GLDM | DependenceVariance |
| F372 |  | GLDM | GrayLevelNonUniformity |
| F373 |  | GLDM | GrayLevelVariance |
| F374 |  | GLDM | HighGrayLevelEmphasis |
| F375 |  | GLDM | LargeDependenceEmphasis |
| F376 |  | GLDM | LargeDependenceHighGrayLevelEmphasis |
| F377 |  | GLDM | LargeDependenceLowGrayLevelEmphasis |
| F378 |  | GLDM | LowGrayLevelEmphasis |
| F379 |  | GLDM | SmallDependenceEmphasis |
| F380 |  | GLDM | SmallDependenceHighGrayLevelEmphasis |
| F381 |  | GLDM | SmallDependenceLowGrayLevelEmphasis |
| F382 |  | NGTDM | Busyness |
| F383 |  | NGTDM | Coarseness |
| F384 |  | NGTDM | Complexity |
| F385 |  | NGTDM | Contrast |
| F386 |  | NGTDM | Strength |
| F387 | LoG (sigma.4.0.mm.3D) | Firstorder | 10Percentile |
| F388 |  | Firstorder | 90Percentile |
| F389 |  | Firstorder | Energy |
| F390 |  | Firstorder | Entropy |
| F391 |  | Firstorder | InterquartileRange |
| F392 |  | Firstorder | Kurtosis |
| F393 |  | Firstorder | Maximum |
| F394 |  | Firstorder | MeanAbsoluteDeviation |
| F395 |  | Firstorder | Mean |
| F396 |  | Firstorder | Median |
| F397 |  | Firstorder | Minimum |
| F398 |  | Firstorder | Range |
| F399 |  | Firstorder | RobustMeanAbsoluteDeviation |
| F400 |  | Firstorder | RootMeanSquared |
| F401 |  | Firstorder | Skewness |
| F402 |  | Firstorder | TotalEnergy |
| F403 |  | Firstorder | Uniformity |
| F404 |  | Firstorder | Variance |
| F405 |  | GLCM | Autocorrelation |
| F406 |  | GLCM | ClusterProminence |
| F407 |  | GLCM | ClusterShade |
| F408 |  | GLCM | ClusterTendency |
| F409 |  | GLCM | Contrast |
| F410 |  | GLCM | Correlation |
| F411 |  | GLCM | DifferenceAverage |
| F412 |  | GLCM | DifferenceEntropy |
| F413 |  | GLCM | DifferenceVariance |
| F414 |  | GLCM | Id |
| F415 |  | GLCM | Idm |
| F416 |  | GLCM | Idmn |
| F417 |  | GLCM | Idn |
| F418 |  | GLCM | Imc1 |
| F419 |  | GLCM | Imc2 |
| F420 |  | GLCM | InverseVariance |
| F421 |  | GLCM | JointAverage |
| F422 |  | GLCM | JointEnergy |
| F423 |  | GLCM | JointEntropy |
| F424 |  | GLCM | MCC |
| F425 |  | GLCM | MaximumProbability |
| F426 |  | GLCM | SumAverage |
| F427 |  | GLCM | SumEntropy |
| F428 |  | GLCM | SumSquares |
| F429 |  | GLRLM | GrayLevelNonUniformity |
| F430 |  | GLRLM | GrayLevelNonUniformityNormalized |
| F431 |  | GLRLM | GrayLevelVariance |
| F432 |  | GLRLM | HighGrayLevelRunEmphasis |
| F433 |  | GLRLM | LongRunEmphasis |
| F434 |  | GLRLM | LongRunHighGrayLevelEmphasis |
| F435 |  | GLRLM | LongRunLowGrayLevelEmphasis |
| F436 |  | GLRLM | LowGrayLevelRunEmphasis |
| F437 |  | GLRLM | RunEntropy |
| F438 |  | GLRLM | RunLengthNonUniformity |
| F439 |  | GLRLM | RunLengthNonUniformityNormalized |
| F440 |  | GLRLM | RunPercentage |
| F441 |  | GLRLM | RunVariance |
| F442 |  | GLRLM | ShortRunEmphasis |
| F443 |  | GLRLM | ShortRunHighGrayLevelEmphasis |
| F444 |  | GLRLM | ShortRunLowGrayLevelEmphasis |
| F445 |  | GLSZM | GrayLevelNonUniformity |
| F446 |  | GLSZM | GrayLevelNonUniformityNormalized |
| F447 |  | GLSZM | GrayLevelVariance |
| F448 |  | GLSZM | HighGrayLevelZoneEmphasis |
| F449 |  | GLSZM | LargeAreaEmphasis |
| F450 |  | GLSZM | LargeAreaHighGrayLevelEmphasis |
| F451 |  | GLSZM | LargeAreaLowGrayLevelEmphasis |
| F452 |  | GLSZM | LowGrayLevelZoneEmphasis |
| F453 |  | GLSZM | SizeZoneNonUniformity |
| F454 |  | GLSZM | SizeZoneNonUniformityNormalized |
| F455 |  | GLSZM | SmallAreaEmphasis |
| F456 |  | GLSZM | SmallAreaHighGrayLevelEmphasis |
| F457 |  | GLSZM | SmallAreaLowGrayLevelEmphasis |
| F458 |  | GLSZM | ZoneEntropy |
| F459 |  | GLSZM | ZonePercentage |
| F460 |  | GLSZM | ZoneVariance |
| F461 |  | GLDM | DependenceEntropy |
| F462 |  | GLDM | DependenceNonUniformity |
| F463 |  | GLDM | DependenceNonUniformityNormalized |
| F464 |  | GLDM | DependenceVariance |
| F465 |  | GLDM | GrayLevelNonUniformity |
| F466 |  | GLDM | GrayLevelVariance |
| F467 |  | GLDM | HighGrayLevelEmphasis |
| F468 |  | GLDM | LargeDependenceEmphasis |
| F469 |  | GLDM | LargeDependenceHighGrayLevelEmphasis |
| F470 |  | GLDM | LargeDependenceLowGrayLevelEmphasis |
| F471 |  | GLDM | LowGrayLevelEmphasis |
| F472 |  | GLDM | SmallDependenceEmphasis |
| F473 |  | GLDM | SmallDependenceHighGrayLevelEmphasis |
| F474 |  | GLDM | SmallDependenceLowGrayLevelEmphasis |
| F475 |  | NGTDM | Busyness |
| F476 |  | NGTDM | Coarseness |
| F477 |  | NGTDM | Complexity |
| F478 |  | NGTDM | Contrast |
| F479 |  | NGTDM | Strength |
| F480 | LoG (sigma.5.0.mm.3D) | Firstorder | 10Percentile |
| F481 |  | Firstorder | 90Percentile |
| F482 |  | Firstorder | Energy |
| F483 |  | Firstorder | Entropy |
| F484 |  | Firstorder | InterquartileRange |
| F485 |  | Firstorder | Kurtosis |
| F486 |  | Firstorder | Maximum |
| F487 |  | Firstorder | MeanAbsoluteDeviation |
| F488 |  | Firstorder | Mean |
| F489 |  | Firstorder | Median |
| F490 |  | Firstorder | Minimum |
| F491 |  | Firstorder | Range |
| F492 |  | Firstorder | RobustMeanAbsoluteDeviation |
| F493 |  | Firstorder | RootMeanSquared |
| F494 |  | Firstorder | Skewness |
| F495 |  | Firstorder | TotalEnergy |
| F496 |  | Firstorder | Uniformity |
| F497 |  | Firstorder | Variance |
| F498 |  | GLCM | Autocorrelation |
| F499 |  | GLCM | ClusterProminence |
| F500 |  | GLCM | ClusterShade |
| F501 |  | GLCM | ClusterTendency |
| F502 |  | GLCM | Contrast |
| F503 |  | GLCM | Correlation |
| F504 |  | GLCM | DifferenceAverage |
| F505 |  | GLCM | DifferenceEntropy |
| F506 |  | GLCM | DifferenceVariance |
| F507 |  | GLCM | Id |
| F508 |  | GLCM | Idm |
| F509 |  | GLCM | Idmn |
| F510 |  | GLCM | Idn |
| F511 |  | GLCM | Imc1 |
| F512 |  | GLCM | Imc2 |
| F513 |  | GLCM | InverseVariance |
| F514 |  | GLCM | JointAverage |
| F515 |  | GLCM | JointEnergy |
| F516 |  | GLCM | JointEntropy |
| F517 |  | GLCM | MCC |
| F518 |  | GLCM | MaximumProbability |
| F519 |  | GLCM | SumAverage |
| F520 |  | GLCM | SumEntropy |
| F521 |  | GLCM | SumSquares |
| F522 |  | GLRLM | GrayLevelNonUniformity |
| F523 |  | GLRLM | GrayLevelNonUniformityNormalized |
| F524 |  | GLRLM | GrayLevelVariance |
| F525 |  | GLRLM | HighGrayLevelRunEmphasis |
| F526 |  | GLRLM | LongRunEmphasis |
| F527 |  | GLRLM | LongRunHighGrayLevelEmphasis |
| F528 |  | GLRLM | LongRunLowGrayLevelEmphasis |
| F529 |  | GLRLM | LowGrayLevelRunEmphasis |
| F530 |  | GLRLM | RunEntropy |
| F531 |  | GLRLM | RunLengthNonUniformity |
| F532 |  | GLRLM | RunLengthNonUniformityNormalized |
| F533 |  | GLRLM | RunPercentage |
| F534 |  | GLRLM | RunVariance |
| F535 |  | GLRLM | ShortRunEmphasis |
| F536 |  | GLRLM | ShortRunHighGrayLevelEmphasis |
| F537 |  | GLRLM | ShortRunLowGrayLevelEmphasis |
| F538 |  | GLSZM | GrayLevelNonUniformity |
| F539 |  | GLSZM | GrayLevelNonUniformityNormalized |
| F540 |  | GLSZM | GrayLevelVariance |
| F541 |  | GLSZM | HighGrayLevelZoneEmphasis |
| F542 |  | GLSZM | LargeAreaEmphasis |
| F543 |  | GLSZM | LargeAreaHighGrayLevelEmphasis |
| F544 |  | GLSZM | LargeAreaLowGrayLevelEmphasis |
| F545 |  | GLSZM | LowGrayLevelZoneEmphasis |
| F546 |  | GLSZM | SizeZoneNonUniformity |
| F547 |  | GLSZM | SizeZoneNonUniformityNormalized |
| F548 |  | GLSZM | SmallAreaEmphasis |
| F549 |  | GLSZM | SmallAreaHighGrayLevelEmphasis |
| F550 |  | GLSZM | SmallAreaLowGrayLevelEmphasis |
| F551 |  | GLSZM | ZoneEntropy |
| F552 |  | GLSZM | ZonePercentage |
| F553 |  | GLSZM | ZoneVariance |
| F554 |  | GLDM | DependenceEntropy |
| F555 |  | GLDM | DependenceNonUniformity |
| F556 |  | GLDM | DependenceNonUniformityNormalized |
| F557 |  | GLDM | DependenceVariance |
| F558 |  | GLDM | GrayLevelNonUniformity |
| F559 |  | GLDM | GrayLevelVariance |
| F560 |  | GLDM | HighGrayLevelEmphasis |
| F561 |  | GLDM | LargeDependenceEmphasis |
| F562 |  | GLDM | LargeDependenceHighGrayLevelEmphasis |
| F563 |  | GLDM | LargeDependenceLowGrayLevelEmphasis |
| F564 |  | GLDM | LowGrayLevelEmphasis |
| F565 |  | GLDM | SmallDependenceEmphasis |
| F566 |  | GLDM | SmallDependenceHighGrayLevelEmphasis |
| F567 |  | GLDM | SmallDependenceLowGrayLevelEmphasis |
| F568 |  | NGTDM | Busyness |
| F569 |  | NGTDM | Coarseness |
| F570 |  | NGTDM | Complexity |
| F571 |  | NGTDM | Contrast |
| F572 |  | NGTDM | Strength |
| F573 | Wavelet-LLH | Firstorder | 10Percentile |
| F574 |  | Firstorder | 90Percentile |
| F575 |  | Firstorder | Energy |
| F576 |  | Firstorder | Entropy |
| F577 |  | Firstorder | InterquartileRange |
| F578 |  | Firstorder | Kurtosis |
| F579 |  | Firstorder | Maximum |
| F580 |  | Firstorder | MeanAbsoluteDeviation |
| F581 |  | Firstorder | Mean |
| F582 |  | Firstorder | Median |
| F583 |  | Firstorder | Minimum |
| F584 |  | Firstorder | Range |
| F585 |  | Firstorder | RobustMeanAbsoluteDeviation |
| F586 |  | Firstorder | RootMeanSquared |
| F587 |  | Firstorder | Skewness |
| F588 |  | Firstorder | TotalEnergy |
| F589 |  | Firstorder | Uniformity |
| F590 |  | Firstorder | Variance |
| F591 |  | GLCM | Autocorrelation |
| F592 |  | GLCM | ClusterProminence |
| F593 |  | GLCM | ClusterShade |
| F594 |  | GLCM | ClusterTendency |
| F595 |  | GLCM | Contrast |
| F596 |  | GLCM | Correlation |
| F597 |  | GLCM | DifferenceAverage |
| F598 |  | GLCM | DifferenceEntropy |
| F599 |  | GLCM | DifferenceVariance |
| F600 |  | GLCM | Id |
| F601 |  | GLCM | Idm |
| F602 |  | GLCM | Idmn |
| F603 |  | GLCM | Idn |
| F604 |  | GLCM | Imc1 |
| F605 |  | GLCM | Imc2 |
| F606 |  | GLCM | InverseVariance |
| F607 |  | GLCM | JointAverage |
| F608 |  | GLCM | JointEnergy |
| F609 |  | GLCM | JointEntropy |
| F610 |  | GLCM | MCC |
| F611 |  | GLCM | MaximumProbability |
| F612 |  | GLCM | SumAverage |
| F613 |  | GLCM | SumEntropy |
| F614 |  | GLCM | SumSquares |
| F615 |  | GLRLM | GrayLevelNonUniformity |
| F616 |  | GLRLM | GrayLevelNonUniformityNormalized |
| F617 |  | GLRLM | GrayLevelVariance |
| F618 |  | GLRLM | HighGrayLevelRunEmphasis |
| F619 |  | GLRLM | LongRunEmphasis |
| F620 |  | GLRLM | LongRunHighGrayLevelEmphasis |
| F621 |  | GLRLM | LongRunLowGrayLevelEmphasis |
| F622 |  | GLRLM | LowGrayLevelRunEmphasis |
| F623 |  | GLRLM | RunEntropy |
| F624 |  | GLRLM | RunLengthNonUniformity |
| F625 |  | GLRLM | RunLengthNonUniformityNormalized |
| F626 |  | GLRLM | RunPercentage |
| F627 |  | GLRLM | RunVariance |
| F628 |  | GLRLM | ShortRunEmphasis |
| F629 |  | GLRLM | ShortRunHighGrayLevelEmphasis |
| F630 |  | GLRLM | ShortRunLowGrayLevelEmphasis |
| F631 |  | GLSZM | GrayLevelNonUniformity |
| F632 |  | GLSZM | GrayLevelNonUniformityNormalized |
| F633 |  | GLSZM | GrayLevelVariance |
| F634 |  | GLSZM | HighGrayLevelZoneEmphasis |
| F635 |  | GLSZM | LargeAreaEmphasis |
| F636 |  | GLSZM | LargeAreaHighGrayLevelEmphasis |
| F637 |  | GLSZM | LargeAreaLowGrayLevelEmphasis |
| F638 |  | GLSZM | LowGrayLevelZoneEmphasis |
| F639 |  | GLSZM | SizeZoneNonUniformity |
| F640 |  | GLSZM | SizeZoneNonUniformityNormalized |
| F641 |  | GLSZM | SmallAreaEmphasis |
| F642 |  | GLSZM | SmallAreaHighGrayLevelEmphasis |
| F643 |  | GLSZM | SmallAreaLowGrayLevelEmphasis |
| F644 |  | GLSZM | ZoneEntropy |
| F645 |  | GLSZM | ZonePercentage |
| F646 |  | GLSZM | ZoneVariance |
| F647 |  | GLDM | DependenceEntropy |
| F648 |  | GLDM | DependenceNonUniformity |
| F649 |  | GLDM | DependenceNonUniformityNormalized |
| F650 |  | GLDM | DependenceVariance |
| F651 |  | GLDM | GrayLevelNonUniformity |
| F652 |  | GLDM | GrayLevelVariance |
| F653 |  | GLDM | HighGrayLevelEmphasis |
| F654 |  | GLDM | LargeDependenceEmphasis |
| F655 |  | GLDM | LargeDependenceHighGrayLevelEmphasis |
| F656 |  | GLDM | LargeDependenceLowGrayLevelEmphasis |
| F657 |  | GLDM | LowGrayLevelEmphasis |
| F658 |  | GLDM | SmallDependenceEmphasis |
| F659 |  | GLDM | SmallDependenceHighGrayLevelEmphasis |
| F660 |  | GLDM | SmallDependenceLowGrayLevelEmphasis |
| F661 |  | NGTDM | Busyness |
| F662 |  | NGTDM | Coarseness |
| F663 |  | NGTDM | Complexity |
| F664 |  | NGTDM | Contrast |
| F665 |  | NGTDM | Strength |
| F666 | wavelet-LHL | Firstorder | 10Percentile |
| F667 |  | Firstorder | 90Percentile |
| F668 |  | Firstorder | Energy |
| F669 |  | Firstorder | Entropy |
| F670 |  | Firstorder | InterquartileRange |
| F671 |  | Firstorder | Kurtosis |
| F672 |  | Firstorder | Maximum |
| F673 |  | Firstorder | MeanAbsoluteDeviation |
| F674 |  | Firstorder | Mean |
| F675 |  | Firstorder | Median |
| F676 |  | Firstorder | Minimum |
| F677 |  | Firstorder | Range |
| F678 |  | Firstorder | RobustMeanAbsoluteDeviation |
| F679 |  | Firstorder | RootMeanSquared |
| F680 |  | Firstorder | Skewness |
| F681 |  | Firstorder | TotalEnergy |
| F682 |  | Firstorder | Uniformity |
| F683 |  | Firstorder | Variance |
| F684 |  | GLCM | Autocorrelation |
| F685 |  | GLCM | ClusterProminence |
| F686 |  | GLCM | ClusterShade |
| F687 |  | GLCM | ClusterTendency |
| F688 |  | GLCM | Contrast |
| F689 |  | GLCM | Correlation |
| F690 |  | GLCM | DifferenceAverage |
| F691 |  | GLCM | DifferenceEntropy |
| F692 |  | GLCM | DifferenceVariance |
| F693 |  | GLCM | Id |
| F694 |  | GLCM | Idm |
| F695 |  | GLCM | Idmn |
| F696 |  | GLCM | Idn |
| F697 |  | GLCM | Imc1 |
| F698 |  | GLCM | Imc2 |
| F699 |  | GLCM | InverseVariance |
| F700 |  | GLCM | JointAverage |
| F701 |  | GLCM | JointEnergy |
| F702 |  | GLCM | JointEntropy |
| F703 |  | GLCM | MCC |
| F704 |  | GLCM | MaximumProbability |
| F705 |  | GLCM | SumAverage |
| F706 |  | GLCM | SumEntropy |
| F707 |  | GLCM | SumSquares |
| F708 |  | GLRLM | GrayLevelNonUniformity |
| F709 |  | GLRLM | GrayLevelNonUniformityNormalized |
| F710 |  | GLRLM | GrayLevelVariance |
| F711 |  | GLRLM | HighGrayLevelRunEmphasis |
| F712 |  | GLRLM | LongRunEmphasis |
| F713 |  | GLRLM | LongRunHighGrayLevelEmphasis |
| F714 |  | GLRLM | LongRunLowGrayLevelEmphasis |
| F715 |  | GLRLM | LowGrayLevelRunEmphasis |
| F716 |  | GLRLM | RunEntropy |
| F717 |  | GLRLM | RunLengthNonUniformity |
| F718 |  | GLRLM | RunLengthNonUniformityNormalized |
| F719 |  | GLRLM | RunPercentage |
| F720 |  | GLRLM | RunVariance |
| F721 |  | GLRLM | ShortRunEmphasis |
| F722 |  | GLRLM | ShortRunHighGrayLevelEmphasis |
| F723 |  | GLRLM | ShortRunLowGrayLevelEmphasis |
| F724 |  | GLSZM | GrayLevelNonUniformity |
| F725 |  | GLSZM | GrayLevelNonUniformityNormalized |
| F726 |  | GLSZM | GrayLevelVariance |
| F727 |  | GLSZM | HighGrayLevelZoneEmphasis |
| F728 |  | GLSZM | LargeAreaEmphasis |
| F729 |  | GLSZM | LargeAreaHighGrayLevelEmphasis |
| F730 |  | GLSZM | LargeAreaLowGrayLevelEmphasis |
| F731 |  | GLSZM | LowGrayLevelZoneEmphasis |
| F732 |  | GLSZM | SizeZoneNonUniformity |
| F733 |  | GLSZM | SizeZoneNonUniformityNormalized |
| F734 |  | GLSZM | SmallAreaEmphasis |
| F735 |  | GLSZM | SmallAreaHighGrayLevelEmphasis |
| F736 |  | GLSZM | SmallAreaLowGrayLevelEmphasis |
| F737 |  | GLSZM | ZoneEntropy |
| F738 |  | GLSZM | ZonePercentage |
| F739 |  | GLSZM | ZoneVariance |
| F740 |  | GLDM | DependenceEntropy |
| F741 |  | GLDM | DependenceNonUniformity |
| F742 |  | GLDM | DependenceNonUniformityNormalized |
| F743 |  | GLDM | DependenceVariance |
| F744 |  | GLDM | GrayLevelNonUniformity |
| F745 |  | GLDM | GrayLevelVariance |
| F746 |  | GLDM | HighGrayLevelEmphasis |
| F747 |  | GLDM | LargeDependenceEmphasis |
| F748 |  | GLDM | LargeDependenceHighGrayLevelEmphasis |
| F749 |  | GLDM | LargeDependenceLowGrayLevelEmphasis |
| F750 |  | GLDM | LowGrayLevelEmphasis |
| F751 |  | GLDM | SmallDependenceEmphasis |
| F752 |  | GLDM | SmallDependenceHighGrayLevelEmphasis |
| F753 |  | GLDM | SmallDependenceLowGrayLevelEmphasis |
| F754 |  | NGTDM | Busyness |
| F755 |  | NGTDM | Coarseness |
| F756 |  | NGTDM | Complexity |
| F757 |  | NGTDM | Contrast |
| F758 |  | NGTDM | Strength |
| F759 | Wavelet-LHH | Firstorder | 10Percentile |
| F760 |  | Firstorder | 90Percentile |
| F761 |  | Firstorder | Energy |
| F762 |  | Firstorder | Entropy |
| F763 |  | Firstorder | InterquartileRange |
| F764 |  | Firstorder | Kurtosis |
| F765 |  | Firstorder | Maximum |
| F766 |  | Firstorder | MeanAbsoluteDeviation |
| F767 |  | Firstorder | Mean |
| F768 |  | Firstorder | Median |
| F769 |  | Firstorder | Minimum |
| F770 |  | Firstorder | Range |
| F771 |  | Firstorder | RobustMeanAbsoluteDeviation |
| F772 |  | Firstorder | RootMeanSquared |
| F773 |  | Firstorder | Skewness |
| F774 |  | Firstorder | TotalEnergy |
| F775 |  | Firstorder | Uniformity |
| F776 |  | Firstorder | Variance |
| F777 |  | GLCM | Autocorrelation |
| F778 |  | GLCM | ClusterProminence |
| F779 |  | GLCM | ClusterShade |
| F780 |  | GLCM | ClusterTendency |
| F781 |  | GLCM | Contrast |
| F782 |  | GLCM | Correlation |
| F783 |  | GLCM | DifferenceAverage |
| F784 |  | GLCM | DifferenceEntropy |
| F785 |  | GLCM | DifferenceVariance |
| F786 |  | GLCM | Id |
| F787 |  | GLCM | Idm |
| F788 |  | GLCM | Idmn |
| F789 |  | GLCM | Idn |
| F790 |  | GLCM | Imc1 |
| F791 |  | GLCM | Imc2 |
| F792 |  | GLCM | InverseVariance |
| F793 |  | GLCM | JointAverage |
| F794 |  | GLCM | JointEnergy |
| F795 |  | GLCM | JointEntropy |
| F796 |  | GLCM | MCC |
| F797 |  | GLCM | MaximumProbability |
| F798 |  | GLCM | SumAverage |
| F799 |  | GLCM | SumEntropy |
| F800 |  | GLCM | SumSquares |
| F801 |  | GLRLM | GrayLevelNonUniformity |
| F802 |  | GLRLM | GrayLevelNonUniformityNormalized |
| F803 |  | GLRLM | GrayLevelVariance |
| F804 |  | GLRLM | HighGrayLevelRunEmphasis |
| F805 |  | GLRLM | LongRunEmphasis |
| F806 |  | GLRLM | LongRunHighGrayLevelEmphasis |
| F807 |  | GLRLM | LongRunLowGrayLevelEmphasis |
| F808 |  | GLRLM | LowGrayLevelRunEmphasis |
| F809 |  | GLRLM | RunEntropy |
| F810 |  | GLRLM | RunLengthNonUniformity |
| F811 |  | GLRLM | RunLengthNonUniformityNormalized |
| F812 |  | GLRLM | RunPercentage |
| F813 |  | GLRLM | RunVariance |
| F814 |  | GLRLM | ShortRunEmphasis |
| F815 |  | GLRLM | ShortRunHighGrayLevelEmphasis |
| F816 |  | GLRLM | ShortRunLowGrayLevelEmphasis |
| F817 |  | GLSZM | GrayLevelNonUniformity |
| F818 |  | GLSZM | GrayLevelNonUniformityNormalized |
| F819 |  | GLSZM | GrayLevelVariance |
| F820 |  | GLSZM | HighGrayLevelZoneEmphasis |
| F821 |  | GLSZM | LargeAreaEmphasis |
| F822 |  | GLSZM | LargeAreaHighGrayLevelEmphasis |
| F823 |  | GLSZM | LargeAreaLowGrayLevelEmphasis |
| F824 |  | GLSZM | LowGrayLevelZoneEmphasis |
| F825 |  | GLSZM | SizeZoneNonUniformity |
| F826 |  | GLSZM | SizeZoneNonUniformityNormalized |
| F827 |  | GLSZM | SmallAreaEmphasis |
| F828 |  | GLSZM | SmallAreaHighGrayLevelEmphasis |
| F829 |  | GLSZM | SmallAreaLowGrayLevelEmphasis |
| F830 |  | GLSZM | ZoneEntropy |
| F831 |  | GLSZM | ZonePercentage |
| F832 |  | GLSZM | ZoneVariance |
| F833 |  | GLDM | DependenceEntropy |
| F834 |  | GLDM | DependenceNonUniformity |
| F835 |  | GLDM | DependenceNonUniformityNormalized |
| F836 |  | GLDM | DependenceVariance |
| F837 |  | GLDM | GrayLevelNonUniformity |
| F838 |  | GLDM | GrayLevelVariance |
| F839 |  | GLDM | HighGrayLevelEmphasis |
| F840 |  | GLDM | LargeDependenceEmphasis |
| F841 |  | GLDM | LargeDependenceHighGrayLevelEmphasis |
| F842 |  | GLDM | LargeDependenceLowGrayLevelEmphasis |
| F843 |  | GLDM | LowGrayLevelEmphasis |
| F844 |  | GLDM | SmallDependenceEmphasis |
| F845 |  | GLDM | SmallDependenceHighGrayLevelEmphasis |
| F846 |  | GLDM | SmallDependenceLowGrayLevelEmphasis |
| F847 |  | NGTDM | Busyness |
| F848 |  | NGTDM | Coarseness |
| F849 |  | NGTDM | Complexity |
| F850 |  | NGTDM | Contrast |
| F851 |  | NGTDM | Strength |
| F852 | Wavelet-HLL | Firstorder | 10Percentile |
| F853 |  | Firstorder | 90Percentile |
| F854 |  | Firstorder | Energy |
| F855 |  | Firstorder | Entropy |
| F856 |  | Firstorder | InterquartileRange |
| F857 |  | Firstorder | Kurtosis |
| F858 |  | Firstorder | Maximum |
| F859 |  | Firstorder | MeanAbsoluteDeviation |
| F860 |  | Firstorder | Mean |
| F861 |  | Firstorder | Median |
| F862 |  | Firstorder | Minimum |
| F863 |  | Firstorder | Range |
| F864 |  | Firstorder | RobustMeanAbsoluteDeviation |
| F865 |  | Firstorder | RootMeanSquared |
| F866 |  | Firstorder | Skewness |
| F867 |  | Firstorder | TotalEnergy |
| F868 |  | Firstorder | Uniformity |
| F869 |  | Firstorder | Variance |
| F870 |  | GLCM | Autocorrelation |
| F871 |  | GLCM | ClusterProminence |
| F872 |  | GLCM | ClusterShade |
| F873 |  | GLCM | ClusterTendency |
| F874 |  | GLCM | Contrast |
| F875 |  | GLCM | Correlation |
| F876 |  | GLCM | DifferenceAverage |
| F877 |  | GLCM | DifferenceEntropy |
| F878 |  | GLCM | DifferenceVariance |
| F879 |  | GLCM | Id |
| F880 |  | GLCM | Idm |
| F881 |  | GLCM | Idmn |
| F882 |  | GLCM | Idn |
| F883 |  | GLCM | Imc1 |
| F884 |  | GLCM | Imc2 |
| F885 |  | GLCM | InverseVariance |
| F886 |  | GLCM | JointAverage |
| F887 |  | GLCM | JointEnergy |
| F888 |  | GLCM | JointEntropy |
| F889 |  | GLCM | MCC |
| F890 |  | GLCM | MaximumProbability |
| F891 |  | GLCM | SumAverage |
| F892 |  | GLCM | SumEntropy |
| F893 |  | GLCM | SumSquares |
| F894 |  | GLRLM | GrayLevelNonUniformity |
| F895 |  | GLRLM | GrayLevelNonUniformityNormalized |
| F896 |  | GLRLM | GrayLevelVariance |
| F897 |  | GLRLM | HighGrayLevelRunEmphasis |
| F898 |  | GLRLM | LongRunEmphasis |
| F899 |  | GLRLM | LongRunHighGrayLevelEmphasis |
| F900 |  | GLRLM | LongRunLowGrayLevelEmphasis |
| F901 |  | GLRLM | LowGrayLevelRunEmphasis |
| F902 |  | GLRLM | RunEntropy |
| F903 |  | GLRLM | RunLengthNonUniformity |
| F904 |  | GLRLM | RunLengthNonUniformityNormalized |
| F905 |  | GLRLM | RunPercentage |
| F906 |  | GLRLM | RunVariance |
| F907 |  | GLRLM | ShortRunEmphasis |
| F908 |  | GLRLM | ShortRunHighGrayLevelEmphasis |
| F909 |  | GLRLM | ShortRunLowGrayLevelEmphasis |
| F910 |  | GLSZM | GrayLevelNonUniformity |
| F911 |  | GLSZM | GrayLevelNonUniformityNormalized |
| F912 |  | GLSZM | GrayLevelVariance |
| F913 |  | GLSZM | HighGrayLevelZoneEmphasis |
| F914 |  | GLSZM | LargeAreaEmphasis |
| F915 |  | GLSZM | LargeAreaHighGrayLevelEmphasis |
| F916 |  | GLSZM | LargeAreaLowGrayLevelEmphasis |
| F917 |  | GLSZM | LowGrayLevelZoneEmphasis |
| F918 |  | GLSZM | SizeZoneNonUniformity |
| F919 |  | GLSZM | SizeZoneNonUniformityNormalized |
| F920 |  | GLSZM | SmallAreaEmphasis |
| F921 |  | GLSZM | SmallAreaHighGrayLevelEmphasis |
| F922 |  | GLSZM | SmallAreaLowGrayLevelEmphasis |
| F923 |  | GLSZM | ZoneEntropy |
| F924 |  | GLSZM | ZonePercentage |
| F925 |  | GLSZM | ZoneVariance |
| F926 |  | GLDM | DependenceEntropy |
| F927 |  | GLDM | DependenceNonUniformity |
| F928 |  | GLDM | DependenceNonUniformityNormalized |
| F929 |  | GLDM | DependenceVariance |
| F930 |  | GLDM | GrayLevelNonUniformity |
| F931 |  | GLDM | GrayLevelVariance |
| F932 |  | GLDM | HighGrayLevelEmphasis |
| F933 |  | GLDM | LargeDependenceEmphasis |
| F934 |  | GLDM | LargeDependenceHighGrayLevelEmphasis |
| F935 |  | GLDM | LargeDependenceLowGrayLevelEmphasis |
| F936 |  | GLDM | LowGrayLevelEmphasis |
| F937 |  | GLDM | SmallDependenceEmphasis |
| F938 |  | GLDM | SmallDependenceHighGrayLevelEmphasis |
| F939 |  | GLDM | SmallDependenceLowGrayLevelEmphasis |
| F940 |  | NGTDM | Busyness |
| F941 |  | NGTDM | Coarseness |
| F942 |  | NGTDM | Complexity |
| F943 |  | NGTDM | Contrast |
| F944 |  | NGTDM | Strength |
| F945 | Wavelet-HLH | Firstorder | 10Percentile |
| F946 |  | Firstorder | 90Percentile |
| F947 |  | Firstorder | Energy |
| F948 |  | Firstorder | Entropy |
| F949 |  | Firstorder | InterquartileRange |
| F950 |  | Firstorder | Kurtosis |
| F951 |  | Firstorder | Maximum |
| F952 |  | Firstorder | MeanAbsoluteDeviation |
| F953 |  | Firstorder | Mean |
| F954 |  | Firstorder | Median |
| F955 |  | Firstorder | Minimum |
| F956 |  | Firstorder | Range |
| F957 |  | Firstorder | RobustMeanAbsoluteDeviation |
| F958 |  | Firstorder | RootMeanSquared |
| F959 |  | Firstorder | Skewness |
| F960 |  | Firstorder | TotalEnergy |
| F961 |  | Firstorder | Uniformity |
| F962 |  | Firstorder | Variance |
| F963 |  | GLCM | Autocorrelation |
| F964 |  | GLCM | ClusterProminence |
| F965 |  | GLCM | ClusterShade |
| F966 |  | GLCM | ClusterTendency |
| F967 |  | GLCM | Contrast |
| F968 |  | GLCM | Correlation |
| F969 |  | GLCM | DifferenceAverage |
| F970 |  | GLCM | DifferenceEntropy |
| F971 |  | GLCM | DifferenceVariance |
| F972 |  | GLCM | Id |
| F973 |  | GLCM | Idm |
| F974 |  | GLCM | Idmn |
| F975 |  | GLCM | Idn |
| F976 |  | GLCM | Imc1 |
| F977 |  | GLCM | Imc2 |
| F978 |  | GLCM | InverseVariance |
| F979 |  | GLCM | JointAverage |
| F980 |  | GLCM | JointEnergy |
| F981 |  | GLCM | JointEntropy |
| F982 |  | GLCM | MCC |
| F983 |  | GLCM | MaximumProbability |
| F984 |  | GLCM | SumAverage |
| F985 |  | GLCM | SumEntropy |
| F986 |  | GLCM | SumSquares |
| F987 |  | GLRLM | GrayLevelNonUniformity |
| F988 |  | GLRLM | GrayLevelNonUniformityNormalized |
| F989 |  | GLRLM | GrayLevelVariance |
| F990 |  | GLRLM | HighGrayLevelRunEmphasis |
| F991 |  | GLRLM | LongRunEmphasis |
| F992 |  | GLRLM | LongRunHighGrayLevelEmphasis |
| F993 |  | GLRLM | LongRunLowGrayLevelEmphasis |
| F994 |  | GLRLM | LowGrayLevelRunEmphasis |
| F995 |  | GLRLM | RunEntropy |
| F996 |  | GLRLM | RunLengthNonUniformity |
| F997 |  | GLRLM | RunLengthNonUniformityNormalized |
| F998 |  | GLRLM | RunPercentage |
| F999 |  | GLRLM | RunVariance |
| F1000 |  | GLRLM | ShortRunEmphasis |
| F1001 |  | GLRLM | ShortRunHighGrayLevelEmphasis |
| F1002 |  | GLRLM | ShortRunLowGrayLevelEmphasis |
| F1003 |  | GLSZM | GrayLevelNonUniformity |
| F1004 |  | GLSZM | GrayLevelNonUniformityNormalized |
| F1005 |  | GLSZM | GrayLevelVariance |
| F1006 |  | GLSZM | HighGrayLevelZoneEmphasis |
| F1007 |  | GLSZM | LargeAreaEmphasis |
| F1008 |  | GLSZM | LargeAreaHighGrayLevelEmphasis |
| F1009 |  | GLSZM | LargeAreaLowGrayLevelEmphasis |
| F1010 |  | GLSZM | LowGrayLevelZoneEmphasis |
| F1011 |  | GLSZM | SizeZoneNonUniformity |
| F1012 |  | GLSZM | SizeZoneNonUniformityNormalized |
| F1013 |  | GLSZM | SmallAreaEmphasis |
| F1014 |  | GLSZM | SmallAreaHighGrayLevelEmphasis |
| F1015 |  | GLSZM | SmallAreaLowGrayLevelEmphasis |
| F1016 |  | GLSZM | ZoneEntropy |
| F1017 |  | GLSZM | ZonePercentage |
| F1018 |  | GLSZM | ZoneVariance |
| F1019 |  | GLDM | DependenceEntropy |
| F1020 |  | GLDM | DependenceNonUniformity |
| F1021 |  | GLDM | DependenceNonUniformityNormalized |
| F1022 |  | GLDM | DependenceVariance |
| F1023 |  | GLDM | GrayLevelNonUniformity |
| F1024 |  | GLDM | GrayLevelVariance |
| F1025 |  | GLDM | HighGrayLevelEmphasis |
| F1026 |  | GLDM | LargeDependenceEmphasis |
| F1027 |  | GLDM | LargeDependenceHighGrayLevelEmphasis |
| F1028 |  | GLDM | LargeDependenceLowGrayLevelEmphasis |
| F1029 |  | GLDM | LowGrayLevelEmphasis |
| F1030 |  | GLDM | SmallDependenceEmphasis |
| F1031 |  | GLDM | SmallDependenceHighGrayLevelEmphasis |
| F1032 |  | GLDM | SmallDependenceLowGrayLevelEmphasis |
| F1033 |  | NGTDM | Busyness |
| F1034 |  | NGTDM | Coarseness |
| F1035 |  | NGTDM | Complexity |
| F1036 |  | NGTDM | Contrast |
| F1037 |  | NGTDM | Strength |
| F1038 | Wavelet-HHL | Firstorder | 10Percentile |
| F1039 |  | Firstorder | 90Percentile |
| F1040 |  | Firstorder | Energy |
| F1041 |  | Firstorder | Entropy |
| F1042 |  | Firstorder | InterquartileRange |
| F1043 |  | Firstorder | Kurtosis |
| F1044 |  | Firstorder | Maximum |
| F1045 |  | Firstorder | MeanAbsoluteDeviation |
| F1046 |  | Firstorder | Mean |
| F1047 |  | Firstorder | Median |
| F1048 |  | Firstorder | Minimum |
| F1049 |  | Firstorder | Range |
| F1050 |  | Firstorder | RobustMeanAbsoluteDeviation |
| F1051 |  | Firstorder | RootMeanSquared |
| F1052 |  | Firstorder | Skewness |
| F1053 |  | Firstorder | TotalEnergy |
| F1054 |  | Firstorder | Uniformity |
| F1055 |  | Firstorder | Variance |
| F1056 |  | GLCM | Autocorrelation |
| F1057 |  | GLCM | ClusterProminence |
| F1058 |  | GLCM | ClusterShade |
| F1059 |  | GLCM | ClusterTendency |
| F1060 |  | GLCM | Contrast |
| F1061 |  | GLCM | Correlation |
| F1062 |  | GLCM | DifferenceAverage |
| F1063 |  | GLCM | DifferenceEntropy |
| F1064 |  | GLCM | DifferenceVariance |
| F1065 |  | GLCM | Id |
| F1066 |  | GLCM | Idm |
| F1067 |  | GLCM | Idmn |
| F1068 |  | GLCM | Idn |
| F1069 |  | GLCM | Imc1 |
| F1070 |  | GLCM | Imc2 |
| F1071 |  | GLCM | InverseVariance |
| F1072 |  | GLCM | JointAverage |
| F1073 |  | GLCM | JointEnergy |
| F1074 |  | GLCM | JointEntropy |
| F1075 |  | GLCM | MCC |
| F1076 |  | GLCM | MaximumProbability |
| F1077 |  | GLCM | SumAverage |
| F1078 |  | GLCM | SumEntropy |
| F1079 |  | GLCM | SumSquares |
| F1080 |  | GLRLM | GrayLevelNonUniformity |
| F1081 |  | GLRLM | GrayLevelNonUniformityNormalized |
| F1082 |  | GLRLM | GrayLevelVariance |
| F1083 |  | GLRLM | HighGrayLevelRunEmphasis |
| F1084 |  | GLRLM | LongRunEmphasis |
| F1085 |  | GLRLM | LongRunHighGrayLevelEmphasis |
| F1086 |  | GLRLM | LongRunLowGrayLevelEmphasis |
| F1087 |  | GLRLM | LowGrayLevelRunEmphasis |
| F1088 |  | GLRLM | RunEntropy |
| F1089 |  | GLRLM | RunLengthNonUniformity |
| F1090 |  | GLRLM | RunLengthNonUniformityNormalized |
| F1091 |  | GLRLM | RunPercentage |
| F1092 |  | GLRLM | RunVariance |
| F1093 |  | GLRLM | ShortRunEmphasis |
| F1094 |  | GLRLM | ShortRunHighGrayLevelEmphasis |
| F1095 |  | GLRLM | ShortRunLowGrayLevelEmphasis |
| F1096 |  | GLSZM | GrayLevelNonUniformity |
| F1097 |  | GLSZM | GrayLevelNonUniformityNormalized |
| F1098 |  | GLSZM | GrayLevelVariance |
| F1099 |  | GLSZM | HighGrayLevelZoneEmphasis |
| F1100 |  | GLSZM | LargeAreaEmphasis |
| F1101 |  | GLSZM | LargeAreaHighGrayLevelEmphasis |
| F1102 |  | GLSZM | LargeAreaLowGrayLevelEmphasis |
| F1103 |  | GLSZM | LowGrayLevelZoneEmphasis |
| F1104 |  | GLSZM | SizeZoneNonUniformity |
| F1105 |  | GLSZM | SizeZoneNonUniformityNormalized |
| F1106 |  | GLSZM | SmallAreaEmphasis |
| F1107 |  | GLSZM | SmallAreaHighGrayLevelEmphasis |
| F1108 |  | GLSZM | SmallAreaLowGrayLevelEmphasis |
| F1109 |  | GLSZM | ZoneEntropy |
| F1110 |  | GLSZM | ZonePercentage |
| F1111 |  | GLSZM | ZoneVariance |
| F1112 |  | GLDM | DependenceEntropy |
| F1113 |  | GLDM | DependenceNonUniformity |
| F1114 |  | GLDM | DependenceNonUniformityNormalized |
| F1115 |  | GLDM | DependenceVariance |
| F1116 |  | GLDM | GrayLevelNonUniformity |
| F1117 |  | GLDM | GrayLevelVariance |
| F1118 |  | GLDM | HighGrayLevelEmphasis |
| F1119 |  | GLDM | LargeDependenceEmphasis |
| F1120 |  | GLDM | LargeDependenceHighGrayLevelEmphasis |
| F1121 |  | GLDM | LargeDependenceLowGrayLevelEmphasis |
| F1122 |  | GLDM | LowGrayLevelEmphasis |
| F1123 |  | GLDM | SmallDependenceEmphasis |
| F1124 |  | GLDM | SmallDependenceHighGrayLevelEmphasis |
| F1125 |  | GLDM | SmallDependenceLowGrayLevelEmphasis |
| F1126 |  | NGTDM | Busyness |
| F1127 |  | NGTDM | Coarseness |
| F1128 |  | NGTDM | Complexity |
| F1129 |  | NGTDM | Contrast |
| F1130 |  | NGTDM | Strength |
| F1131 | Wavelet-HHH | Firstorder | 10Percentile |
| F1132 |  | Firstorder | 90Percentile |
| F1133 |  | Firstorder | Energy |
| F1134 |  | Firstorder | Entropy |
| F1135 |  | Firstorder | InterquartileRange |
| F1136 |  | Firstorder | Kurtosis |
| F1137 |  | Firstorder | Maximum |
| F1138 |  | Firstorder | MeanAbsoluteDeviation |
| F1139 |  | Firstorder | Mean |
| F1140 |  | Firstorder | Median |
| F1141 |  | Firstorder | Minimum |
| F1142 |  | Firstorder | Range |
| F1143 |  | Firstorder | RobustMeanAbsoluteDeviation |
| F1144 |  | Firstorder | RootMeanSquared |
| F1145 |  | Firstorder | Skewness |
| F1146 |  | Firstorder | TotalEnergy |
| F1147 |  | Firstorder | Uniformity |
| F1148 |  | Firstorder | Variance |
| F1149 |  | GLCM | Autocorrelation |
| F1150 |  | GLCM | ClusterProminence |
| F1151 |  | GLCM | ClusterShade |
| F1152 |  | GLCM | ClusterTendency |
| F1153 |  | GLCM | Contrast |
| F1154 |  | GLCM | Correlation |
| F1155 |  | GLCM | DifferenceAverage |
| F1156 |  | GLCM | DifferenceEntropy |
| F1157 |  | GLCM | DifferenceVariance |
| F1158 |  | GLCM | Id |
| F1159 |  | GLCM | Idm |
| F1160 |  | GLCM | Idmn |
| F1161 |  | GLCM | Idn |
| F1162 |  | GLCM | Imc1 |
| F1163 |  | GLCM | Imc2 |
| F1164 |  | GLCM | InverseVariance |
| F1165 |  | GLCM | JointAverage |
| F1166 |  | GLCM | JointEnergy |
| F1167 |  | GLCM | JointEntropy |
| F1168 |  | GLCM | MCC |
| F1169 |  | GLCM | MaximumProbability |
| F1170 |  | GLCM | SumAverage |
| F1171 |  | GLCM | SumEntropy |
| F1172 |  | GLCM | SumSquares |
| F1173 |  | GLRLM | GrayLevelNonUniformity |
| F1174 |  | GLRLM | GrayLevelNonUniformityNormalized |
| F1175 |  | GLRLM | GrayLevelVariance |
| F1176 |  | GLRLM | HighGrayLevelRunEmphasis |
| F1177 |  | GLRLM | LongRunEmphasis |
| F1178 |  | GLRLM | LongRunHighGrayLevelEmphasis |
| F1179 |  | GLRLM | LongRunLowGrayLevelEmphasis |
| F1180 |  | GLRLM | LowGrayLevelRunEmphasis |
| F1181 |  | GLRLM | RunEntropy |
| F1182 |  | GLRLM | RunLengthNonUniformity |
| F1183 |  | GLRLM | RunLengthNonUniformityNormalized |
| F1184 |  | GLRLM | RunPercentage |
| F1185 |  | GLRLM | RunVariance |
| F1186 |  | GLRLM | ShortRunEmphasis |
| F1187 |  | GLRLM | ShortRunHighGrayLevelEmphasis |
| F1188 |  | GLRLM | ShortRunLowGrayLevelEmphasis |
| F1189 |  | GLSZM | GrayLevelNonUniformity |
| F1190 |  | GLSZM | GrayLevelNonUniformityNormalized |
| F1191 |  | GLSZM | GrayLevelVariance |
| F1192 |  | GLSZM | HighGrayLevelZoneEmphasis |
| F1193 |  | GLSZM | LargeAreaEmphasis |
| F1194 |  | GLSZM | LargeAreaHighGrayLevelEmphasis |
| F1195 |  | GLSZM | LargeAreaLowGrayLevelEmphasis |
| F1196 |  | GLSZM | LowGrayLevelZoneEmphasis |
| F1197 |  | GLSZM | SizeZoneNonUniformity |
| F1198 |  | GLSZM | SizeZoneNonUniformityNormalized |
| F1199 |  | GLSZM | SmallAreaEmphasis |
| F1200 |  | GLSZM | SmallAreaHighGrayLevelEmphasis |
| F1201 |  | GLSZM | SmallAreaLowGrayLevelEmphasis |
| F1202 |  | GLSZM | ZoneEntropy |
| F1203 |  | GLSZM | ZonePercentage |
| F1204 |  | GLSZM | ZoneVariance |
| F1205 |  | GLDM | DependenceEntropy |
| F1206 |  | GLDM | DependenceNonUniformity |
| F1207 |  | GLDM | DependenceNonUniformityNormalized |
| F1208 |  | GLDM | DependenceVariance |
| F1209 |  | GLDM | GrayLevelNonUniformity |
| F1210 |  | GLDM | GrayLevelVariance |
| F1211 |  | GLDM | HighGrayLevelEmphasis |
| F1212 |  | GLDM | LargeDependenceEmphasis |
| F1213 |  | GLDM | LargeDependenceHighGrayLevelEmphasis |
| F1214 |  | GLDM | LargeDependenceLowGrayLevelEmphasis |
| F1215 |  | GLDM | LowGrayLevelEmphasis |
| F1216 |  | GLDM | SmallDependenceEmphasis |
| F1217 |  | GLDM | SmallDependenceHighGrayLevelEmphasis |
| F1218 |  | GLDM | SmallDependenceLowGrayLevelEmphasis |
| F1219 |  | NGTDM | Busyness |
| F1220 |  | NGTDM | Coarseness |
| F1221 |  | NGTDM | Complexity |
| F1222 |  | NGTDM | Contrast |
| F1223 |  | NGTDM | Strength |
| F1224 | Wavelet-LLL | Firstorder | 10Percentile |
| F1225 |  | Firstorder | 90Percentile |
| F1226 |  | Firstorder | Energy |
| F1227 |  | Firstorder | Entropy |
| F1228 |  | Firstorder | InterquartileRange |
| F1229 |  | Firstorder | Kurtosis |
| F1230 |  | Firstorder | Maximum |
| F1231 |  | Firstorder | MeanAbsoluteDeviation |
| F1232 |  | Firstorder | Mean |
| F1233 |  | Firstorder | Median |
| F1234 |  | Firstorder | Minimum |
| F1235 |  | Firstorder | Range |
| F1236 |  | Firstorder | RobustMeanAbsoluteDeviation |
| F1237 |  | Firstorder | RootMeanSquared |
| F1238 |  | Firstorder | Skewness |
| F1239 |  | Firstorder | TotalEnergy |
| F1240 |  | Firstorder | Uniformity |
| F1241 |  | Firstorder | Variance |
| F1242 |  | GLCM | Autocorrelation |
| F1243 |  | GLCM | ClusterProminence |
| F1244 |  | GLCM | ClusterShade |
| F1245 |  | GLCM | ClusterTendency |
| F1246 |  | GLCM | Contrast |
| F1247 |  | GLCM | Correlation |
| F1248 |  | GLCM | DifferenceAverage |
| F1249 |  | GLCM | DifferenceEntropy |
| F1250 |  | GLCM | DifferenceVariance |
| F1251 |  | GLCM | Id |
| F1252 |  | GLCM | Idm |
| F1253 |  | GLCM | Idmn |
| F1254 |  | GLCM | Idn |
| F1255 |  | GLCM | Imc1 |
| F1256 |  | GLCM | Imc2 |
| F1257 |  | GLCM | InverseVariance |
| F1258 |  | GLCM | JointAverage |
| F1259 |  | GLCM | JointEnergy |
| F1260 |  | GLCM | JointEntropy |
| F1261 |  | GLCM | MCC |
| F1262 |  | GLCM | MaximumProbability |
| F1263 |  | GLCM | SumAverage |
| F1264 |  | GLCM | SumEntropy |
| F1265 |  | GLCM | SumSquares |
| F1266 |  | GLRLM | GrayLevelNonUniformity |
| F1267 |  | GLRLM | GrayLevelNonUniformityNormalized |
| F1268 |  | GLRLM | GrayLevelVariance |
| F1269 |  | GLRLM | HighGrayLevelRunEmphasis |
| F1270 |  | GLRLM | LongRunEmphasis |
| F1271 |  | GLRLM | LongRunHighGrayLevelEmphasis |
| F1272 |  | GLRLM | LongRunLowGrayLevelEmphasis |
| F1273 |  | GLRLM | LowGrayLevelRunEmphasis |
| F1274 |  | GLRLM | RunEntropy |
| F1275 |  | GLRLM | RunLengthNonUniformity |
| F1276 |  | GLRLM | RunLengthNonUniformityNormalized |
| F1277 |  | GLRLM | RunPercentage |
| F1278 |  | GLRLM | RunVariance |
| F1279 |  | GLRLM | ShortRunEmphasis |
| F1280 |  | GLRLM | ShortRunHighGrayLevelEmphasis |
| F1281 |  | GLRLM | ShortRunLowGrayLevelEmphasis |
| F1282 |  | GLSZM | GrayLevelNonUniformity |
| F1283 |  | GLSZM | GrayLevelNonUniformityNormalized |
| F1284 |  | GLSZM | GrayLevelVariance |
| F1285 |  | GLSZM | HighGrayLevelZoneEmphasis |
| F1286 |  | GLSZM | LargeAreaEmphasis |
| F1287 |  | GLSZM | LargeAreaHighGrayLevelEmphasis |
| F1288 |  | GLSZM | LargeAreaLowGrayLevelEmphasis |
| F1289 |  | GLSZM | LowGrayLevelZoneEmphasis |
| F1290 |  | GLSZM | SizeZoneNonUniformity |
| F1291 |  | GLSZM | SizeZoneNonUniformityNormalized |
| F1292 |  | GLSZM | SmallAreaEmphasis |
| F1293 |  | GLSZM | SmallAreaHighGrayLevelEmphasis |
| F1294 |  | GLSZM | SmallAreaLowGrayLevelEmphasis |
| F1295 |  | GLSZM | ZoneEntropy |
| F1296 |  | GLSZM | ZonePercentage |
| F1297 |  | GLSZM | ZoneVariance |
| F1298 |  | GLDM | DependenceEntropy |
| F1299 |  | GLDM | DependenceNonUniformity |
| F1300 |  | GLDM | DependenceNonUniformityNormalized |
| F1301 |  | GLDM | DependenceVariance |
| F1302 |  | GLDM | GrayLevelNonUniformity |
| F1303 |  | GLDM | GrayLevelVariance |
| F1304 |  | GLDM | HighGrayLevelEmphasis |
| F1305 |  | GLDM | LargeDependenceEmphasis |
| F1306 |  | GLDM | LargeDependenceHighGrayLevelEmphasis |
| F1307 |  | GLDM | LargeDependenceLowGrayLevelEmphasis |
| F1308 |  | GLDM | LowGrayLevelEmphasis |
| F1309 |  | GLDM | SmallDependenceEmphasis |
| F1310 |  | GLDM | SmallDependenceHighGrayLevelEmphasis |
| F1311 |  | GLDM | SmallDependenceLowGrayLevelEmphasis |
| F1312 |  | NGTDM | Busyness |
| F1313 |  | NGTDM | Coarseness |
| F1314 |  | NGTDM | Complexity |
| F1315 |  | NGTDM | Contrast |
| F1316 |  | NGTDM | Strength |
| F1317 | Square | Firstorder | 10Percentile |
| F1318 |  | Firstorder | 90Percentile |
| F1319 |  | Firstorder | Energy |
| F1320 |  | Firstorder | Entropy |
| F1321 |  | Firstorder | InterquartileRange |
| F1322 |  | Firstorder | Kurtosis |
| F1323 |  | Firstorder | Maximum |
| F1324 |  | Firstorder | MeanAbsoluteDeviation |
| F1325 |  | Firstorder | Mean |
| F1326 |  | Firstorder | Median |
| F1327 |  | Firstorder | Minimum |
| F1328 |  | Firstorder | Range |
| F1329 |  | Firstorder | RobustMeanAbsoluteDeviation |
| F1330 |  | Firstorder | RootMeanSquared |
| F1331 |  | Firstorder | Skewness |
| F1332 |  | Firstorder | TotalEnergy |
| F1333 |  | Firstorder | Uniformity |
| F1334 |  | Firstorder | Variance |
| F1335 |  | GLCM | Autocorrelation |
| F1336 |  | GLCM | ClusterProminence |
| F1337 |  | GLCM | ClusterShade |
| F1338 |  | GLCM | ClusterTendency |
| F1339 |  | GLCM | Contrast |
| F1340 |  | GLCM | Correlation |
| F1341 |  | GLCM | DifferenceAverage |
| F1342 |  | GLCM | DifferenceEntropy |
| F1343 |  | GLCM | DifferenceVariance |
| F1344 |  | GLCM | Id |
| F1345 |  | GLCM | Idm |
| F1346 |  | GLCM | Idmn |
| F1347 |  | GLCM | Idn |
| F1348 |  | GLCM | Imc1 |
| F1349 |  | GLCM | Imc2 |
| F1350 |  | GLCM | InverseVariance |
| F1351 |  | GLCM | JointAverage |
| F1352 |  | GLCM | JointEnergy |
| F1353 |  | GLCM | JointEntropy |
| F1354 |  | GLCM | MCC |
| F1355 |  | GLCM | MaximumProbability |
| F1356 |  | GLCM | SumAverage |
| F1357 |  | GLCM | SumEntropy |
| F1358 |  | GLCM | SumSquares |
| F1359 |  | GLRLM | GrayLevelNonUniformity |
| F1360 |  | GLRLM | GrayLevelNonUniformityNormalized |
| F1361 |  | GLRLM | GrayLevelVariance |
| F1362 |  | GLRLM | HighGrayLevelRunEmphasis |
| F1363 |  | GLRLM | LongRunEmphasis |
| F1364 |  | GLRLM | LongRunHighGrayLevelEmphasis |
| F1365 |  | GLRLM | LongRunLowGrayLevelEmphasis |
| F1366 |  | GLRLM | LowGrayLevelRunEmphasis |
| F1367 |  | GLRLM | RunEntropy |
| F1368 |  | GLRLM | RunLengthNonUniformity |
| F1369 |  | GLRLM | RunLengthNonUniformityNormalized |
| F1370 |  | GLRLM | RunPercentage |
| F1371 |  | GLRLM | RunVariance |
| F1372 |  | GLRLM | ShortRunEmphasis |
| F1373 |  | GLRLM | ShortRunHighGrayLevelEmphasis |
| F1374 |  | GLRLM | ShortRunLowGrayLevelEmphasis |
| F1375 |  | GLSZM | GrayLevelNonUniformity |
| F1376 |  | GLSZM | GrayLevelNonUniformityNormalized |
| F1377 |  | GLSZM | GrayLevelVariance |
| F1378 |  | GLSZM | HighGrayLevelZoneEmphasis |
| F1379 |  | GLSZM | LargeAreaEmphasis |
| F1380 |  | GLSZM | LargeAreaHighGrayLevelEmphasis |
| F1381 |  | GLSZM | LargeAreaLowGrayLevelEmphasis |
| F1382 |  | GLSZM | LowGrayLevelZoneEmphasis |
| F1383 |  | GLSZM | SizeZoneNonUniformity |
| F1384 |  | GLSZM | SizeZoneNonUniformityNormalized |
| F1385 |  | GLSZM | SmallAreaEmphasis |
| F1386 |  | GLSZM | SmallAreaHighGrayLevelEmphasis |
| F1387 |  | GLSZM | SmallAreaLowGrayLevelEmphasis |
| F1388 |  | GLSZM | ZoneEntropy |
| F1389 |  | GLSZM | ZonePercentage |
| F1390 |  | GLSZM | ZoneVariance |
| F1391 |  | GLDM | DependenceEntropy |
| F1392 |  | GLDM | DependenceNonUniformity |
| F1393 |  | GLDM | DependenceNonUniformityNormalized |
| F1394 |  | GLDM | DependenceVariance |
| F1395 |  | GLDM | GrayLevelNonUniformity |
| F1396 |  | GLDM | GrayLevelVariance |
| F1397 |  | GLDM | HighGrayLevelEmphasis |
| F1398 |  | GLDM | LargeDependenceEmphasis |
| F1399 |  | GLDM | LargeDependenceHighGrayLevelEmphasis |
| F1400 |  | GLDM | LargeDependenceLowGrayLevelEmphasis |
| F1401 |  | GLDM | LowGrayLevelEmphasis |
| F1402 |  | GLDM | SmallDependenceEmphasis |
| F1403 |  | GLDM | SmallDependenceHighGrayLevelEmphasis |
| F1404 |  | GLDM | SmallDependenceLowGrayLevelEmphasis |
| F1405 |  | NGTDM | Busyness |
| F1406 |  | NGTDM | Coarseness |
| F1407 |  | NGTDM | Complexity |
| F1408 |  | NGTDM | Contrast |
| F1409 |  | NGTDM | Strength |
| F1410 | SquareRoot | Firstorder | 10Percentile |
| F1411 |  | Firstorder | 90Percentile |
| F1412 |  | Firstorder | Energy |
| F1413 |  | Firstorder | Entropy |
| F1414 |  | Firstorder | InterquartileRange |
| F1415 |  | Firstorder | Kurtosis |
| F1416 |  | Firstorder | Maximum |
| F1417 |  | Firstorder | MeanAbsoluteDeviation |
| F1418 |  | Firstorder | Mean |
| F1419 |  | Firstorder | Median |
| F1420 |  | Firstorder | Minimum |
| F1421 |  | Firstorder | Range |
| F1422 |  | Firstorder | RobustMeanAbsoluteDeviation |
| F1423 |  | Firstorder | RootMeanSquared |
| F1424 |  | Firstorder | Skewness |
| F1425 |  | Firstorder | TotalEnergy |
| F1426 |  | Firstorder | Uniformity |
| F1427 |  | Firstorder | Variance |
| F1428 |  | GLCM | Autocorrelation |
| F1429 |  | GLCM | ClusterProminence |
| F1430 |  | GLCM | ClusterShade |
| F1431 |  | GLCM | ClusterTendency |
| F1432 |  | GLCM | Contrast |
| F1433 |  | GLCM | Correlation |
| F1434 |  | GLCM | DifferenceAverage |
| F1435 |  | GLCM | DifferenceEntropy |
| F1436 |  | GLCM | DifferenceVariance |
| F1437 |  | GLCM | Id |
| F1438 |  | GLCM | Idm |
| F1439 |  | GLCM | Idmn |
| F1440 |  | GLCM | Idn |
| F1441 |  | GLCM | Imc1 |
| F1442 |  | GLCM | Imc2 |
| F1443 |  | GLCM | InverseVariance |
| F1444 |  | GLCM | JointAverage |
| F1445 |  | GLCM | JointEnergy |
| F1446 |  | GLCM | JointEntropy |
| F1447 |  | GLCM | MCC |
| F1448 |  | GLCM | MaximumProbability |
| F1449 |  | GLCM | SumAverage |
| F1450 |  | GLCM | SumEntropy |
| F1451 |  | GLCM | SumSquares |
| F1452 |  | GLRLM | GrayLevelNonUniformity |
| F1453 |  | GLRLM | GrayLevelNonUniformityNormalized |
| F1454 |  | GLRLM | GrayLevelVariance |
| F1455 |  | GLRLM | HighGrayLevelRunEmphasis |
| F1456 |  | GLRLM | LongRunEmphasis |
| F1457 |  | GLRLM | LongRunHighGrayLevelEmphasis |
| F1458 |  | GLRLM | LongRunLowGrayLevelEmphasis |
| F1459 |  | GLRLM | LowGrayLevelRunEmphasis |
| F1460 |  | GLRLM | RunEntropy |
| F1461 |  | GLRLM | RunLengthNonUniformity |
| F1462 |  | GLRLM | RunLengthNonUniformityNormalized |
| F1463 |  | GLRLM | RunPercentage |
| F1464 |  | GLRLM | RunVariance |
| F1465 |  | GLRLM | ShortRunEmphasis |
| F1466 |  | GLRLM | ShortRunHighGrayLevelEmphasis |
| F1467 |  | GLRLM | ShortRunLowGrayLevelEmphasis |
| F1468 |  | GLSZM | GrayLevelNonUniformity |
| F1469 |  | GLSZM | GrayLevelNonUniformityNormalized |
| F1470 |  | GLSZM | GrayLevelVariance |
| F1471 |  | GLSZM | HighGrayLevelZoneEmphasis |
| F1472 |  | GLSZM | LargeAreaEmphasis |
| F1473 |  | GLSZM | LargeAreaHighGrayLevelEmphasis |
| F1474 |  | GLSZM | LargeAreaLowGrayLevelEmphasis |
| F1475 |  | GLSZM | LowGrayLevelZoneEmphasis |
| F1476 |  | GLSZM | SizeZoneNonUniformity |
| F1477 |  | GLSZM | SizeZoneNonUniformityNormalized |
| F1478 |  | GLSZM | SmallAreaEmphasis |
| F1479 |  | GLSZM | SmallAreaHighGrayLevelEmphasis |
| F1480 |  | GLSZM | SmallAreaLowGrayLevelEmphasis |
| F1481 |  | GLSZM | ZoneEntropy |
| F1482 |  | GLSZM | ZonePercentage |
| F1483 |  | GLSZM | ZoneVariance |
| F1484 |  | GLDM | DependenceEntropy |
| F1485 |  | GLDM | DependenceNonUniformity |
| F1486 |  | GLDM | DependenceNonUniformityNormalized |
| F1487 |  | GLDM | DependenceVariance |
| F1488 |  | GLDM | GrayLevelNonUniformity |
| F1489 |  | GLDM | GrayLevelVariance |
| F1490 |  | GLDM | HighGrayLevelEmphasis |
| F1491 |  | GLDM | LargeDependenceEmphasis |
| F1492 |  | GLDM | LargeDependenceHighGrayLevelEmphasis |
| F1493 |  | GLDM | LargeDependenceLowGrayLevelEmphasis |
| F1494 |  | GLDM | LowGrayLevelEmphasis |
| F1495 |  | GLDM | SmallDependenceEmphasis |
| F1496 |  | GLDM | SmallDependenceHighGrayLevelEmphasis |
| F1497 |  | GLDM | SmallDependenceLowGrayLevelEmphasis |
| F1498 |  | NGTDM | Busyness |
| F1499 |  | NGTDM | Coarseness |
| F1500 |  | NGTDM | Complexity |
| F1501 |  | NGTDM | Contrast |
| F1502 |  | NGTDM | Strength |
| F1503 | Logarithm | Firstorder | 10Percentile |
| F1504 |  | Firstorder | 90Percentile |
| F1505 |  | Firstorder | Energy |
| F1506 |  | Firstorder | Entropy |
| F1507 |  | Firstorder | InterquartileRange |
| F1508 |  | Firstorder | Kurtosis |
| F1509 |  | Firstorder | Maximum |
| F1510 |  | Firstorder | MeanAbsoluteDeviation |
| F1511 |  | Firstorder | Mean |
| F1512 |  | Firstorder | Median |
| F1513 |  | Firstorder | Minimum |
| F1514 |  | Firstorder | Range |
| F1515 |  | Firstorder | RobustMeanAbsoluteDeviation |
| F1516 |  | Firstorder | RootMeanSquared |
| F1517 |  | Firstorder | Skewness |
| F1518 |  | Firstorder | TotalEnergy |
| F1519 |  | Firstorder | Uniformity |
| F1520 |  | Firstorder | Variance |
| F1521 |  | GLCM | Autocorrelation |
| F1522 |  | GLCM | ClusterProminence |
| F1523 |  | GLCM | ClusterShade |
| F1524 |  | GLCM | ClusterTendency |
| F1525 |  | GLCM | Contrast |
| F1526 |  | GLCM | Correlation |
| F1527 |  | GLCM | DifferenceAverage |
| F1528 |  | GLCM | DifferenceEntropy |
| F1529 |  | GLCM | DifferenceVariance |
| F1530 |  | GLCM | Id |
| F1531 |  | GLCM | Idm |
| F1532 |  | GLCM | Idmn |
| F1533 |  | GLCM | Idn |
| F1534 |  | GLCM | Imc1 |
| F1535 |  | GLCM | Imc2 |
| F1536 |  | GLCM | InverseVariance |
| F1537 |  | GLCM | JointAverage |
| F1538 |  | GLCM | JointEnergy |
| F1539 |  | GLCM | JointEntropy |
| F1540 |  | GLCM | MCC |
| F1541 |  | GLCM | MaximumProbability |
| F1542 |  | GLCM | SumAverage |
| F1543 |  | GLCM | SumEntropy |
| F1544 |  | GLCM | SumSquares |
| F1545 |  | GLRLM | GrayLevelNonUniformity |
| F1546 |  | GLRLM | GrayLevelNonUniformityNormalized |
| F1547 |  | GLRLM | GrayLevelVariance |
| F1548 |  | GLRLM | HighGrayLevelRunEmphasis |
| F1549 |  | GLRLM | LongRunEmphasis |
| F1550 |  | GLRLM | LongRunHighGrayLevelEmphasis |
| F1551 |  | GLRLM | LongRunLowGrayLevelEmphasis |
| F1552 |  | GLRLM | LowGrayLevelRunEmphasis |
| F1553 |  | GLRLM | RunEntropy |
| F1554 |  | GLRLM | RunLengthNonUniformity |
| F1555 |  | GLRLM | RunLengthNonUniformityNormalized |
| F1556 |  | GLRLM | RunPercentage |
| F1557 |  | GLRLM | RunVariance |
| F1558 |  | GLRLM | ShortRunEmphasis |
| F1559 |  | GLRLM | ShortRunHighGrayLevelEmphasis |
| F1560 |  | GLRLM | ShortRunLowGrayLevelEmphasis |
| F1561 |  | GLSZM | GrayLevelNonUniformity |
| F1562 |  | GLSZM | GrayLevelNonUniformityNormalized |
| F1563 |  | GLSZM | GrayLevelVariance |
| F1564 |  | GLSZM | HighGrayLevelZoneEmphasis |
| F1565 |  | GLSZM | LargeAreaEmphasis |
| F1566 |  | GLSZM | LargeAreaHighGrayLevelEmphasis |
| F1567 |  | GLSZM | LargeAreaLowGrayLevelEmphasis |
| F1568 |  | GLSZM | LowGrayLevelZoneEmphasis |
| F1569 |  | GLSZM | SizeZoneNonUniformity |
| F1570 |  | GLSZM | SizeZoneNonUniformityNormalized |
| F1571 |  | GLSZM | SmallAreaEmphasis |
| F1572 |  | GLSZM | SmallAreaHighGrayLevelEmphasis |
| F1573 |  | GLSZM | SmallAreaLowGrayLevelEmphasis |
| F1574 |  | GLSZM | ZoneEntropy |
| F1575 |  | GLSZM | ZonePercentage |
| F1576 |  | GLSZM | ZoneVariance |
| F1577 |  | GLDM | DependenceEntropy |
| F1578 |  | GLDM | DependenceNonUniformity |
| F1579 |  | GLDM | DependenceNonUniformityNormalized |
| F1580 |  | GLDM | DependenceVariance |
| F1581 |  | GLDM | GrayLevelNonUniformity |
| F1582 |  | GLDM | GrayLevelVariance |
| F1583 |  | GLDM | HighGrayLevelEmphasis |
| F1584 |  | GLDM | LargeDependenceEmphasis |
| F1585 |  | GLDM | LargeDependenceHighGrayLevelEmphasis |
| F1586 |  | GLDM | LargeDependenceLowGrayLevelEmphasis |
| F1587 |  | GLDM | LowGrayLevelEmphasis |
| F1588 |  | GLDM | SmallDependenceEmphasis |
| F1589 |  | GLDM | SmallDependenceHighGrayLevelEmphasis |
| F1590 |  | GLDM | SmallDependenceLowGrayLevelEmphasis |
| F1591 |  | NGTDM | Busyness |
| F1592 |  | NGTDM | Coarseness |
| F1593 |  | NGTDM | Complexity |
| F1594 |  | NGTDM | Contrast |
| F1595 |  | NGTDM | Strength |
| F1596 | Exponential | Firstorder | 10Percentile |
| F1597 |  | Firstorder | 90Percentile |
| F1598 |  | Firstorder | Energy |
| F1599 |  | Firstorder | Entropy |
| F1600 |  | Firstorder | InterquartileRange |
| F1601 |  | Firstorder | Kurtosis |
| F1602 |  | Firstorder | Maximum |
| F1603 |  | Firstorder | MeanAbsoluteDeviation |
| F1604 |  | Firstorder | Mean |
| F1605 |  | Firstorder | Median |
| F1606 |  | Firstorder | Minimum |
| F1607 |  | Firstorder | Range |
| F1608 |  | Firstorder | RobustMeanAbsoluteDeviation |
| F1609 |  | Firstorder | RootMeanSquared |
| F1610 |  | Firstorder | Skewness |
| F1611 |  | Firstorder | TotalEnergy |
| F1612 |  | Firstorder | Uniformity |
| F1613 |  | Firstorder | Variance |
| F1614 |  | GLCM | Autocorrelation |
| F1615 |  | GLCM | ClusterProminence |
| F1616 |  | GLCM | ClusterShade |
| F1617 |  | GLCM | ClusterTendency |
| F1618 |  | GLCM | Contrast |
| F1619 |  | GLCM | Correlation |
| F1620 |  | GLCM | DifferenceAverage |
| F1621 |  | GLCM | DifferenceEntropy |
| F1622 |  | GLCM | DifferenceVariance |
| F1623 |  | GLCM | Id |
| F1624 |  | GLCM | Idm |
| F1625 |  | GLCM | Idmn |
| F1626 |  | GLCM | Idn |
| F1627 |  | GLCM | Imc1 |
| F1628 |  | GLCM | Imc2 |
| F1629 |  | GLCM | InverseVariance |
| F1630 |  | GLCM | JointAverage |
| F1631 |  | GLCM | JointEnergy |
| F1632 |  | GLCM | JointEntropy |
| F1633 |  | GLCM | MCC |
| F1634 |  | GLCM | MaximumProbability |
| F1635 |  | GLCM | SumAverage |
| F1636 |  | GLCM | SumEntropy |
| F1637 |  | GLCM | SumSquares |
| F1638 |  | GLRLM | GrayLevelNonUniformity |
| F1639 |  | GLRLM | GrayLevelNonUniformityNormalized |
| F1640 |  | GLRLM | GrayLevelVariance |
| F1641 |  | GLRLM | HighGrayLevelRunEmphasis |
| F1642 |  | GLRLM | LongRunEmphasis |
| F1643 |  | GLRLM | LongRunHighGrayLevelEmphasis |
| F1644 |  | GLRLM | LongRunLowGrayLevelEmphasis |
| F1645 |  | GLRLM | LowGrayLevelRunEmphasis |
| F1646 |  | GLRLM | RunEntropy |
| F1647 |  | GLRLM | RunLengthNonUniformity |
| F1648 |  | GLRLM | RunLengthNonUniformityNormalized |
| F1649 |  | GLRLM | RunPercentage |
| F1650 |  | GLRLM | RunVariance |
| F1651 |  | GLRLM | ShortRunEmphasis |
| F1652 |  | GLRLM | ShortRunHighGrayLevelEmphasis |
| F1653 |  | GLRLM | ShortRunLowGrayLevelEmphasis |
| F1654 |  | GLSZM | GrayLevelNonUniformity |
| F1655 |  | GLSZM | GrayLevelNonUniformityNormalized |
| F1656 |  | GLSZM | GrayLevelVariance |
| F1657 |  | GLSZM | HighGrayLevelZoneEmphasis |
| F1658 |  | GLSZM | LargeAreaEmphasis |
| F1659 |  | GLSZM | LargeAreaHighGrayLevelEmphasis |
| F1660 |  | GLSZM | LargeAreaLowGrayLevelEmphasis |
| F1661 |  | GLSZM | LowGrayLevelZoneEmphasis |
| F1662 |  | GLSZM | SizeZoneNonUniformity |
| F1663 |  | GLSZM | SizeZoneNonUniformityNormalized |
| F1664 |  | GLSZM | SmallAreaEmphasis |
| F1665 |  | GLSZM | SmallAreaHighGrayLevelEmphasis |
| F1666 |  | GLSZM | SmallAreaLowGrayLevelEmphasis |
| F1667 |  | GLSZM | ZoneEntropy |
| F1668 |  | GLSZM | ZonePercentage |
| F1669 |  | GLSZM | ZoneVariance |
| F1670 |  | GLDM | DependenceEntropy |
| F1671 |  | GLDM | DependenceNonUniformity |
| F1672 |  | GLDM | DependenceNonUniformityNormalized |
| F1673 |  | GLDM | DependenceVariance |
| F1674 |  | GLDM | GrayLevelNonUniformity |
| F1675 |  | GLDM | GrayLevelVariance |
| F1676 |  | GLDM | HighGrayLevelEmphasis |
| F1677 |  | GLDM | LargeDependenceEmphasis |
| F1678 |  | GLDM | LargeDependenceHighGrayLevelEmphasis |
| F1679 |  | GLDM | LargeDependenceLowGrayLevelEmphasis |
| F1680 |  | GLDM | LowGrayLevelEmphasis |
| F1681 |  | GLDM | SmallDependenceEmphasis |
| F1682 |  | GLDM | SmallDependenceHighGrayLevelEmphasis |
| F1683 |  | GLDM | SmallDependenceLowGrayLevelEmphasis |
| F1684 |  | NGTDM | Busyness |
| F1685 |  | NGTDM | Coarseness |
| F1686 |  | NGTDM | Complexity |
| F1687 |  | NGTDM | Contrast |
| F1688 |  | NGTDM | Strength |
| F1689 | Gradient | Firstorder | 10Percentile |
| F1690 |  | Firstorder | 90Percentile |
| F1691 |  | Firstorder | Energy |
| F1692 |  | Firstorder | Entropy |
| F1693 |  | Firstorder | InterquartileRange |
| F1694 |  | Firstorder | Kurtosis |
| F1695 |  | Firstorder | Maximum |
| F1696 |  | Firstorder | MeanAbsoluteDeviation |
| F1697 |  | Firstorder | Mean |
| F1698 |  | Firstorder | Median |
| F1699 |  | Firstorder | Minimum |
| F1700 |  | Firstorder | Range |
| F1701 |  | Firstorder | RobustMeanAbsoluteDeviation |
| F1702 |  | Firstorder | RootMeanSquared |
| F1703 |  | Firstorder | Skewness |
| F1704 |  | Firstorder | TotalEnergy |
| F1705 |  | Firstorder | Uniformity |
| F1706 |  | Firstorder | Variance |
| F1707 |  | GLCM | Autocorrelation |
| F1708 |  | GLCM | ClusterProminence |
| F1709 |  | GLCM | ClusterShade |
| F1710 |  | GLCM | ClusterTendency |
| F1711 |  | GLCM | Contrast |
| F1712 |  | GLCM | Correlation |
| F1713 |  | GLCM | DifferenceAverage |
| F1714 |  | GLCM | DifferenceEntropy |
| F1715 |  | GLCM | DifferenceVariance |
| F1716 |  | GLCM | Id |
| F1717 |  | GLCM | Idm |
| F1718 |  | GLCM | Idmn |
| F1719 |  | GLCM | Idn |
| F1720 |  | GLCM | Imc1 |
| F1721 |  | GLCM | Imc2 |
| F1722 |  | GLCM | InverseVariance |
| F1723 |  | GLCM | JointAverage |
| F1724 |  | GLCM | JointEnergy |
| F1725 |  | GLCM | JointEntropy |
| F1726 |  | GLCM | MCC |
| F1727 |  | GLCM | MaximumProbability |
| F1728 |  | GLCM | SumAverage |
| F1729 |  | GLCM | SumEntropy |
| F1730 |  | GLCM | SumSquares |
| F1731 |  | GLRLM | GrayLevelNonUniformity |
| F1732 |  | GLRLM | GrayLevelNonUniformityNormalized |
| F1733 |  | GLRLM | GrayLevelVariance |
| F1734 |  | GLRLM | HighGrayLevelRunEmphasis |
| F1735 |  | GLRLM | LongRunEmphasis |
| F1736 |  | GLRLM | LongRunHighGrayLevelEmphasis |
| F1737 |  | GLRLM | LongRunLowGrayLevelEmphasis |
| F1738 |  | GLRLM | LowGrayLevelRunEmphasis |
| F1739 |  | GLRLM | RunEntropy |
| F1740 |  | GLRLM | RunLengthNonUniformity |
| F1741 |  | GLRLM | RunLengthNonUniformityNormalized |
| F1742 |  | GLRLM | RunPercentage |
| F1743 |  | GLRLM | RunVariance |
| F1744 |  | GLRLM | ShortRunEmphasis |
| F1745 |  | GLRLM | ShortRunHighGrayLevelEmphasis |
| F1746 |  | GLRLM | ShortRunLowGrayLevelEmphasis |
| F1747 |  | GLSZM | GrayLevelNonUniformity |
| F1748 |  | GLSZM | GrayLevelNonUniformityNormalized |
| F1749 |  | GLSZM | GrayLevelVariance |
| F1750 |  | GLSZM | HighGrayLevelZoneEmphasis |
| F1751 |  | GLSZM | LargeAreaEmphasis |
| F1752 |  | GLSZM | LargeAreaHighGrayLevelEmphasis |
| F1753 |  | GLSZM | LargeAreaLowGrayLevelEmphasis |
| F1754 |  | GLSZM | LowGrayLevelZoneEmphasis |
| F1755 |  | GLSZM | SizeZoneNonUniformity |
| F1756 |  | GLSZM | SizeZoneNonUniformityNormalized |
| F1757 |  | GLSZM | SmallAreaEmphasis |
| F1758 |  | GLSZM | SmallAreaHighGrayLevelEmphasis |
| F1759 |  | GLSZM | SmallAreaLowGrayLevelEmphasis |
| F1760 |  | GLSZM | ZoneEntropy |
| F1761 |  | GLSZM | ZonePercentage |
| F1762 |  | GLSZM | ZoneVariance |
| F1763 |  | GLDM | DependenceEntropy |
| F1764 |  | GLDM | DependenceNonUniformity |
| F1765 |  | GLDM | DependenceNonUniformityNormalized |
| F1766 |  | GLDM | DependenceVariance |
| F1767 |  | GLDM | GrayLevelNonUniformity |
| F1768 |  | GLDM | GrayLevelVariance |
| F1769 |  | GLDM | HighGrayLevelEmphasis |
| F1770 |  | GLDM | LargeDependenceEmphasis |
| F1771 |  | GLDM | LargeDependenceHighGrayLevelEmphasis |
| F1772 |  | GLDM | LargeDependenceLowGrayLevelEmphasis |
| F1773 |  | GLDM | LowGrayLevelEmphasis |
| F1774 |  | GLDM | SmallDependenceEmphasis |
| F1775 |  | GLDM | SmallDependenceHighGrayLevelEmphasis |
| F1776 |  | GLDM | SmallDependenceLowGrayLevelEmphasis |
| F1777 |  | NGTDM | Busyness |
| F1778 |  | NGTDM | Coarseness |
| F1779 |  | NGTDM | Complexity |
| F1780 |  | NGTDM | Contrast |
| F1781 |  | NGTDM | Strength |
| F1782 | LocalBinaryPattern2D | Firstorder | 10Percentile |
| F1783 |  | Firstorder | 90Percentile |
| F1784 |  | Firstorder | Energy |
| F1785 |  | Firstorder | Entropy |
| F1786 |  | Firstorder | InterquartileRange |
| F1787 |  | Firstorder | Kurtosis |
| F1788 |  | Firstorder | Maximum |
| F1789 |  | Firstorder | MeanAbsoluteDeviation |
| F1790 |  | Firstorder | Mean |
| F1791 |  | Firstorder | Median |
| F1792 |  | Firstorder | Minimum |
| F1793 |  | Firstorder | Range |
| F1794 |  | Firstorder | RobustMeanAbsoluteDeviation |
| F1795 |  | Firstorder | RootMeanSquared |
| F1796 |  | Firstorder | Skewness |
| F1797 |  | Firstorder | TotalEnergy |
| F1798 |  | Firstorder | Uniformity |
| F1799 |  | Firstorder | Variance |
| F1800 |  | GLCM | Autocorrelation |
| F1801 |  | GLCM | ClusterProminence |
| F1802 |  | GLCM | ClusterShade |
| F1803 |  | GLCM | ClusterTendency |
| F1804 |  | GLCM | Contrast |
| F1805 |  | GLCM | Correlation |
| F1806 |  | GLCM | DifferenceAverage |
| F1807 |  | GLCM | DifferenceEntropy |
| F1808 |  | GLCM | DifferenceVariance |
| F1809 |  | GLCM | Id |
| F1810 |  | GLCM | Idm |
| F1811 |  | GLCM | Idmn |
| F1812 |  | GLCM | Idn |
| F1813 |  | GLCM | Imc1 |
| F1814 |  | GLCM | Imc2 |
| F1815 |  | GLCM | InverseVariance |
| F1816 |  | GLCM | JointAverage |
| F1817 |  | GLCM | JointEnergy |
| F1818 |  | GLCM | JointEntropy |
| F1819 |  | GLCM | MCC |
| F1820 |  | GLCM | MaximumProbability |
| F1821 |  | GLCM | SumAverage |
| F1822 |  | GLCM | SumEntropy |
| F1823 |  | GLCM | SumSquares |
| F1824 |  | GLRLM | GrayLevelNonUniformity |
| F1825 |  | GLRLM | GrayLevelNonUniformityNormalized |
| F1826 |  | GLRLM | GrayLevelVariance |
| F1827 |  | GLRLM | HighGrayLevelRunEmphasis |
| F1828 |  | GLRLM | LongRunEmphasis |
| F1829 |  | GLRLM | LongRunHighGrayLevelEmphasis |
| F1830 |  | GLRLM | LongRunLowGrayLevelEmphasis |
| F1831 |  | GLRLM | LowGrayLevelRunEmphasis |
| F1832 |  | GLRLM | RunEntropy |
| F1833 |  | GLRLM | RunLengthNonUniformity |
| F1834 |  | GLRLM | RunLengthNonUniformityNormalized |
| F1835 |  | GLRLM | RunPercentage |
| F1836 |  | GLRLM | RunVariance |
| F1837 |  | GLRLM | ShortRunEmphasis |
| F1838 |  | GLRLM | ShortRunHighGrayLevelEmphasis |
| F1839 |  | GLRLM | ShortRunLowGrayLevelEmphasis |
| F1840 |  | GLSZM | GrayLevelNonUniformity |
| F1841 |  | GLSZM | GrayLevelNonUniformityNormalized |
| F1842 |  | GLSZM | GrayLevelVariance |
| F1843 |  | GLSZM | HighGrayLevelZoneEmphasis |
| F1844 |  | GLSZM | LargeAreaEmphasis |
| F1845 |  | GLSZM | LargeAreaHighGrayLevelEmphasis |
| F1846 |  | GLSZM | LargeAreaLowGrayLevelEmphasis |
| F1847 |  | GLSZM | LowGrayLevelZoneEmphasis |
| F1848 |  | GLSZM | SizeZoneNonUniformity |
| F1849 |  | GLSZM | SizeZoneNonUniformityNormalized |
| F1850 |  | GLSZM | SmallAreaEmphasis |
| F1851 |  | GLSZM | SmallAreaHighGrayLevelEmphasis |
| F1852 |  | GLSZM | SmallAreaLowGrayLevelEmphasis |
| F1853 |  | GLSZM | ZoneEntropy |
| F1854 |  | GLSZM | ZonePercentage |
| F1855 |  | GLSZM | ZoneVariance |
| F1856 |  | GLDM | DependenceEntropy |
| F1857 |  | GLDM | DependenceNonUniformity |
| F1858 |  | GLDM | DependenceNonUniformityNormalized |
| F1859 |  | GLDM | DependenceVariance |
| F1860 |  | GLDM | GrayLevelNonUniformity |
| F1861 |  | GLDM | GrayLevelVariance |
| F1862 |  | GLDM | HighGrayLevelEmphasis |
| F1863 |  | GLDM | LargeDependenceEmphasis |
| F1864 |  | GLDM | LargeDependenceHighGrayLevelEmphasis |
| F1865 |  | GLDM | LargeDependenceLowGrayLevelEmphasis |
| F1866 |  | GLDM | LowGrayLevelEmphasis |
| F1867 |  | GLDM | SmallDependenceEmphasis |
| F1868 |  | GLDM | SmallDependenceHighGrayLevelEmphasis |
| F1869 |  | GLDM | SmallDependenceLowGrayLevelEmphasis |
| F1870 |  | NGTDM | Busyness |
| F1871 |  | NGTDM | Coarseness |
| F1872 |  | NGTDM | Complexity |
| F1873 |  | NGTDM | Contrast |
| F1874 |  | NGTDM | Strength |

Abbreviations: GLCM: gray-level co-occurrence matrix; GLRLM: gray-level run-length matrix; GLSZM: gray-level size-zone matrix; GLDM: gray-level dependence matrix; and NGTDM: neighboring gray-tone difference matrix.
